# Cancer cell-secreted IGF2 instigates fibroblasts and bone marrow-derived vascular progenitor cells to promote cancer progression

**DOI:** 10.1038/ncomms14399

**Published:** 2017-02-10

**Authors:** Wen Wen Xu, Bin Li, Xin Yuan Guan, Sookja K. Chung, Yang Wang, Yim Ling Yip, Simon Y. K. Law, Kin Tak Chan, Nikki P. Y. Lee, Kwok Wah Chan, Li Yan Xu, En Min Li, Sai Wah Tsao, Qing-Yu He, Annie L. M. Cheung

**Affiliations:** 1 School of Biomedical Sciences, Li Ka Shing Faculty of Medicine, 21 Sassoon Road, The University of Hong Kong, Pokfulam, Hong Kong SAR, China; 2 The University of Hong Kong-Shenzhen Institute of Research and Innovation (HKU-SIRI), Kejizhong 2nd Rd., Hi-Tech Industrial Park, Nanshan District, Shenzhen 518057, China; 3 Centre for Cancer Research, Li Ka Shing Faculty of Medicine, 21 Sassoon Road, The University of Hong Kong, Pokfulam, Hong Kong SAR, China; 4 Department of Clinical Oncology, Li Ka Shing Faculty of Medicine, 21 Sassoon Road, The University of Hong Kong, Pokfulam, Hong Kong SAR, China; 5 College of Life Science and Technology, Jinan University, 601 West Huangpu Blvd., Guangzhou 510632, China; 6 Department of Surgery, Li Ka Shing Faculty of Medicine, 21 Sassoon Road, The University of Hong Kong, Pokfulam, Hong Kong SAR, China; 7 Department of Pathology, Li Ka Shing Faculty of Medicine, 21 Sassoon Road, The University of Hong Kong, Pokfulam, Hong Kong SAR, China; 8 The Key Laboratory of Molecular Biology for High Cancer Incidence Coastal Chaoshan Area, Shantou University Medical College, 22 Xinling Road, Shantou, 515041 Guangdong, China

## Abstract

Local interactions between cancer cells and stroma can produce systemic effects on distant organs to govern cancer progression. Here we show that IGF2 secreted by inhibitor of differentiation (Id1)-overexpressing oesophageal cancer cells instigates VEGFR1-positive bone marrow cells in the tumour macroenvironment to form pre-metastatic niches at distant sites by increasing VEGF secretion from cancer-associated fibroblasts. Cancer cells are then attracted to the metastatic site via the CXCL5/CXCR2 axis. Bone marrow cells transplanted from nude mice bearing Id1-overexpressing oesophageal tumours enhance tumour growth and metastasis in recipient mice, whereas systemic administration of VEGFR1 antibody abrogates these effects. Mechanistically, IGF2 regulates VEGF in fibroblasts via miR-29c in a p53-dependent manner. Analysis of patient serum samples showed that concurrent elevation of IGF2 and VEGF levels may serve as a prognostic biomarker for oesophageal cancer. These findings suggest that the Id1/IGF2/VEGF/VEGFR1 cascade plays a critical role in tumour-driven pathophysiological processes underlying cancer progression.

## Abstract (web summary)

Cancer cells can affect cancer progression by producing systemic effects. Here, the authors show that IGF2 produced by oesophageal cancer cells increases secretion of VEGF from cancer-associated fibroblasts resulting in systemic mobilization of bone marrow-derived cells and increased metastases.

Cancer has been described as a systemic disease rather than a local phenomenon^1^. Tumour cells not only interact with the stroma in the local environment (tumour microenvironment) but also connect with the body systems (macroenvironment) via blood and lymphatic vessels^2^,^3^. Fibroblasts are the most abundant cell type within the tumour stroma of many cancers, and activation of fibroblasts has been reported to contribute to tumour growth^4^,^5^. In contrast to cancer cells, stromal cells are more genetically stable and thus represent an attractive target for cancer therapy. However, we are still far from fully understanding the complex crosstalk between cancer cells and stroma.

Metastasis is an important process that allows cancer cells to escape from the primary tumour and settle in distant organs. Metastatic cancers are largely incurable and are estimated to account for 90% of mortality from cancer^6^. Although recent studies have shed light on some of the mechanisms of metastasis, the molecular components that mediate the engraftment of tumour cells at these sites have yet to be fully identified.

Tumour growth at both primary and secondary sites requires neovascularization and angiogenesis^7^. Prognosis of patients with esophageal squamous cell carcinoma (ESCC) is correlated with tumour vascularity^8^. The significance of Id (inhibitor of differentiation) proteins in supporting tumour angiogenesis and metastasis was documented in as early as 1999 (ref. 9). Subsequently, upregulation of Id1 was found to be strongly associated with, and functionally contributes to, the development of human cancer^10^,^11^. Moreover, Id1 was reported to have prognostic significance in patients with ESCC^12^,^13^. Our previous studies showed that Id1-overexpression induces ESCC cells to produce and secrete insulin-like growth factor 2 (IGF2), which stimulates cancer cell proliferation in an autocrine manner^14^, and that concurrent high Id1 and IGF2 expression in ESCC is associated with shorter survival^15^. In the present study, we examined whether Id1-induced IGF2 plays any role in tumour angiogenesis and whether it exerts paracrine effects in the tumour microenvironment and tumour macroenvironment to further facilitate cancer progression. We also investigated the cellular crosstalk and molecular signalling in the tumour micro- and macroenvironment in order to obtain a better understanding of cancer progression that may facilitate development of novel systemic therapy. Our results show that IGF2 secreted by Id1-expressing cancer cells not only activates the tumour microenvironment by inducing fibroblasts to secrete vascular endothelial growth factor (VEGF), but this mechanism also instigates the tumour macroenvironment so that bone marrow cells primed by the presence of Id1-expressing tumours can facilitate tumour growth and distant metastatic colonization. These effects can be abolished by systemic administration of VEGFR1 antibody. Furthermore, we reveal that IGF2 regulates VEGF via miR-29c in a p53-dependent manner. These data suggest a critical role for the Id1/IGF2/VEGF/VEGFR cascade in driving oesophageal cancer progression. Furthermore, our study provides evidence to support the potential clinical application of VEGFR1 antibody in the treatment of oesophageal cancer.

## Results

### Id1-induced IGF2 from ESCC cells activates fibroblasts

**Figure 1:**
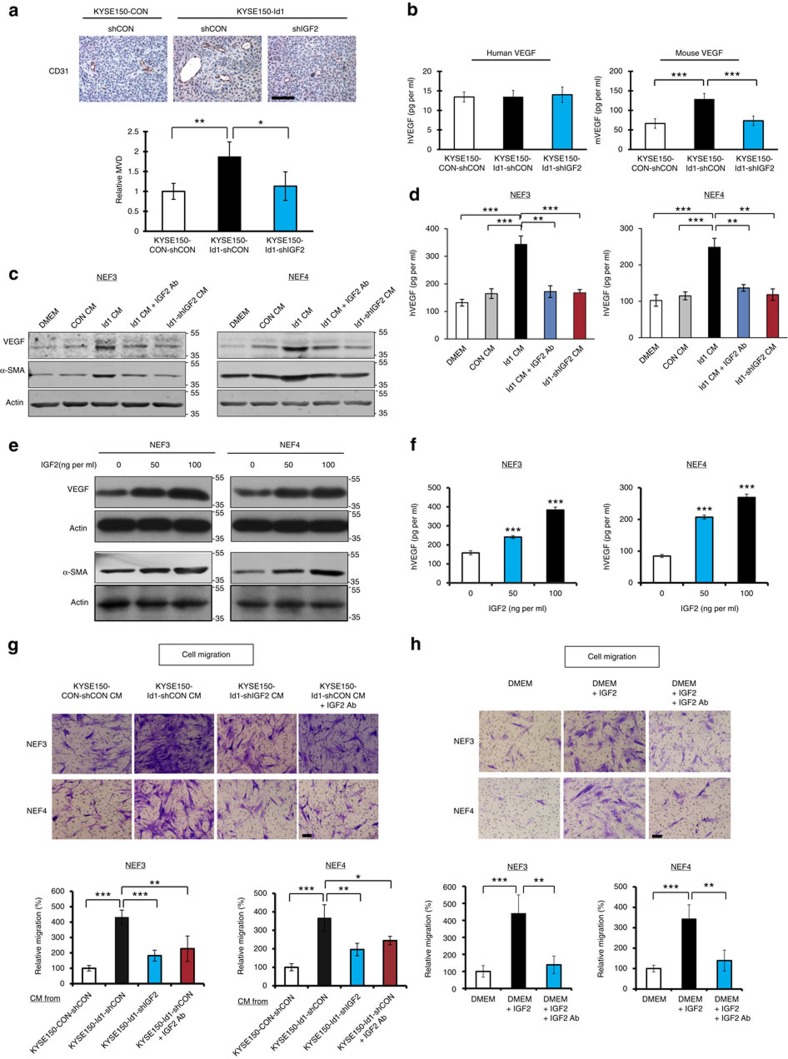
Id1-induced IGF2 activates fibroblasts in a paracrine manner. (**a**) Tumour xenografts established from KYSE150-CON-shCON, KYSE150-Id1-shCON or KYSE150-Id1-shIGF2 ESCC cells were immunostained for CD31 and analysed for microvessel density (female 6–8-week-old nude mice, *n*=3 per group; scale bar, 100 μm). (**b**) Human VEGF (left panel) and mouse VEGF (right panel) concentration in serum of mice bearing Id1-overexpressing, Id1-shIGF2 or control tumours (female 6–8-week-old nude mice, *n*=6 per group) was analysed using ELISA. (**c**,**d**) Expression of VEGF and α-SMA (**c**) and secretion of VEGF (**d**) in fibroblasts fed with conditioned medium (CM) from KYSE150-CON-shCON, KYSE150-Id1-shCON or KYSE150-Id1-shIGF2 cells were assayed using western blot and ELISA, respectively. (**e**,**f**) Expression of VEGF and α-SMA (**e**) and secretion of VEGF (**f**) in fibroblasts treated with recombinant human IGF2. (**g**,**h**) Chemotactic migration of fibroblasts in response to conditioned medium (scale bar, 100 μm) from indicated ESCC cells (**g**) and exogenous IGF2 (**h**).Three biological replicates were performed for *in vitro* assays. Data in bar charts are presented as mean±s.d.; *P<0.05; **P<0.01; ***P<0.001 by Student's *t-*test.

Vascular endothelial growth factor (VEGF)-dependent endothelial cell sprouting is a main mechanism of tumour angiogenesis. To investigate the role of Id1-induced IGF2 on VEGF-mediated tumour angiogenesis, we first compared the microvessel density in subcutaneous tumour xenografts established from KYSE150-Id1-shCON, KYSE150-Id1-shIGF2 and KYSE150-CON-shCON ESCC cells. The results showed higher microvessel density in the Id1-overexpressing tumour xenografts, compared with tumours that expressed Id1-shIGF2 or control vectors (Fig. 1a). We found that although serum concentration of human VEGF in the nude mice was comparable among the three groups, remarkably higher concentration of mouse VEGF was detected in the KYSE150-Id1-shCON group, suggesting that the elevated VEGF was host-derived and likely to be stimulated by Id1-induced IGF2 (Fig. 1b). These results were confirmed using another ESCC cell line, KYSE270 (Supplementary Fig. 1a,b). To determine whether other Id genes could compensate for Id1 in this mechanism, we used specific small interfering RNAs (siRNAs) to knock down Id2, Id3 and Id4, respectively, in ESCC cells and found that manipulating the expression of these Id genes had no effect on the expression of IGF2, thus affirming the specificity of Id1 in this process (Supplementary Fig. 1c).

Next, we investigated the paracrine effect of cancer cell-secreted IGF2 on fibroblasts. Two normal oesophageal fibroblast (NEF) cell lines, designated NEF3 and NEF4 (established from normal oesophageal tissue of patients who underwent surgical resection of primary oesophageal tumour), which had confirmed fibroblastic phenotype (Supplementary Fig. 1d), were used to test the paracrine effects of ESCC cells on fibroblasts. We found that the conditioned medium collected from Id1-overexpressing ESCC cells not only markedly induced NEFs to acquire myofibroblast phenotype characterized by alpha smooth muscle actin (α-SMA) expression, but also stimulated the expression and secretion of VEGF. However, addition of neutralizing antibody against IGF2, or inhibition of IGF2 by shRNA knockdown, failed to recapitulate these effects (Fig. 1c,d). To study whether other angiogenic molecules might be important in this process, we further analysed the fibroblasts treated with conditioned medium from Id1-overexpressing ESCC cells for expression of placental growth factor and fibroblast growth factor. However, we did not observe any change in the expression level of these molecules (Supplementary Fig. 1e).

To directly test whether IGF2 was the main driver of this effect, NEFs were treated with recombinant human IGF2 (rhIGF2), and the results showed a dose-dependent increase of fibroblast VEGF and α-SMA expressions (Fig. 1e), as well as VEGF secretion (Fig. 1f). With respect to functional effects, we found that treatment with conditioned medium from Id1-overexpressing ESCC cells for 24 h induced the migration (Fig. 1g) but not the proliferation (Supplementary Fig. 1f) of fibroblasts, and that the effect was attenuated by IGF2 knockdown in cancer cells, or addition of IGF2-neutralizing antibody to the conditioned medium. The direct involvement of IGF2 in these effects was confirmed by the data showing that rhIGF2 promoted the migration of fibroblasts (Fig. 1h) and that the effect was not due to increased cell proliferation (Supplementary Fig. 1g). Meanwhile, IGF2-activated fibroblasts or fibroblasts co-cultured with Id1-overexpressing ESCC cells were found to exert multifaceted effects on the local tumour microenvironment by inducing the proliferation, migration and tube formation (Supplementary Figs 2a–c and 3) of human umbilical vein endothelial cells, as well as increasing the invasion of ESCC cells (Supplementary Fig. 2d).

Taken together, the results provided the first evidence showing that Id1-induced IGF2 from cancer cells can activate stromal fibroblasts in a paracrine manner, and stimulate them to produce VEGF as well as to adopt a more cancer-promoting phenotype.

### Clinical relevance of serum IGF2 and VEGF expression levels

**Figure 2:**
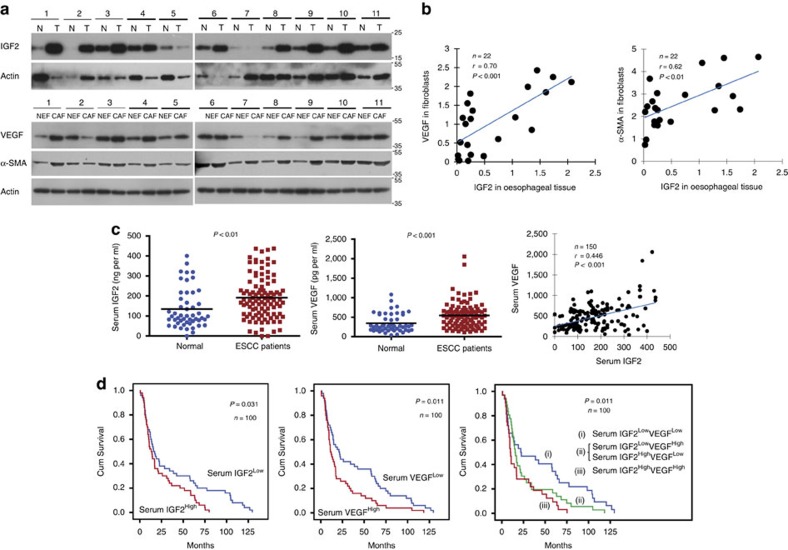
Clinical relevance of tumour IGF2 and stromal VEGF axis. (**a**) Western blot showing expression of IGF2 in ESCC (T) and matched non-tumour specimens (N), as well as expression of VEGF and α-SMA in CAFs and matched NEF from 11 ESCC patients. (**b**) Graphs showing positive correlation between IGF2 expression in oesophageal tissue and expressions of VEGF (left panel) and α-SMA (right panel) in fibroblasts, respectively, in 11 pairs of ESCC and adjacent normal tissues. Correlation was assessed using Pearson's rank correlation coefficient. (**c**) Comparison of serum IGF2 (left panel) and VEGF (middle panel) levels between healthy individuals (*n*=50) and ESCC patients (*n*=100); the data were pooled and a positive correlation was found between serum VEGF and IGF2 levels (*n*=150) (right panel) using unpaired *t*-test. (**d**) Gene expressions were further divided into high and low levels using median expression level as the cut-off point for survival analyses. Kaplan–Meier curves comparing survival outcome of ESCC patients (*n*=100) with high and low serum IGF2 levels (left panel), high and low serum VEGF levels (middle panel), and IGF2 ^High^/VEGF ^High^ and IGF2 ^Low^/VEGF ^Low^ levels (right panel); statistical significance was calculated by log-rank test.

Most studies of tumour VEGF expression do not make a distinction between cancer cells and the stroma. We investigated the correlation between oesophageal tumour IGF2 expression and fibroblast VEGF expression by isolating cancer-associated fibroblasts (CAFs) and NEFs from 11 cases of ESCC and adjacent normal tissues, respectively. Western blot analysis showed that, with the exception of case no. 7, IGF2 expression was elevated in 10/11 (90.9%) of the tumour samples compared with corresponding tumour-adjacent normal tissue (Fig. 2a). Furthermore, there was upregulation of VEGF (except cases no. 2 and 7) and α-SMA (except case no. 2) in CAFs compared with NEFs in 81.8 and 90.9% of the cases, respectively (Fig. 2a). Statistical analysis showed that IGF2 expression in oesophageal tissues was positively correlated with VEGF and α-SMA expressions in the corresponding fibroblasts (Fig. 2b). We also analysed the association between expression levels of Id1 in oesophageal tissues and fibroblast VEGF and found a positive correlation although a statistical significance could not be established (Supplementary Fig. 4a).

To study the clinical relevance of circulating IGF2 and VEGF in ESCC patients, serum IGF2 and VEGF levels in 100 ESCC patients with survival data were determined using ELISA and compared with that of 50 healthy individuals. The IGF2 and VEGF concentrations were found to be higher in ESCC patients, and were positively correlated with each other (Fig. 2c). Moreover, the patients with concomitant high IGF2 and VEGF expression had shorter survival (median survival=10.1 months) than patients with low IGF2 and VEGF expression (median survival=22.3 months) (Fig. 2d). Concurrent elevation of serum IGF2 and VEGF was associated with T3/T4 stages, distant metastasis and advanced pathologic stages (Stages III/IV) (Supplementary Table 1). Multivariate survival analyses showed that combined elevated expression of serum IGF2 and VEGF was an independent unfavourable prognostic indicator for ESCC patients (Supplementary Table 2). In addition, by analysing a dataset containing 53 patients with primary breast tumours in Gene Expression Omnibus (GEO) database, we found a correlation between stromal VEGF level and poor survival and recurrence (Supplementary Fig. 4b).

### miR-29c mediates regulation of IGF2 on VEGF in fibroblasts

**Figure 3:**
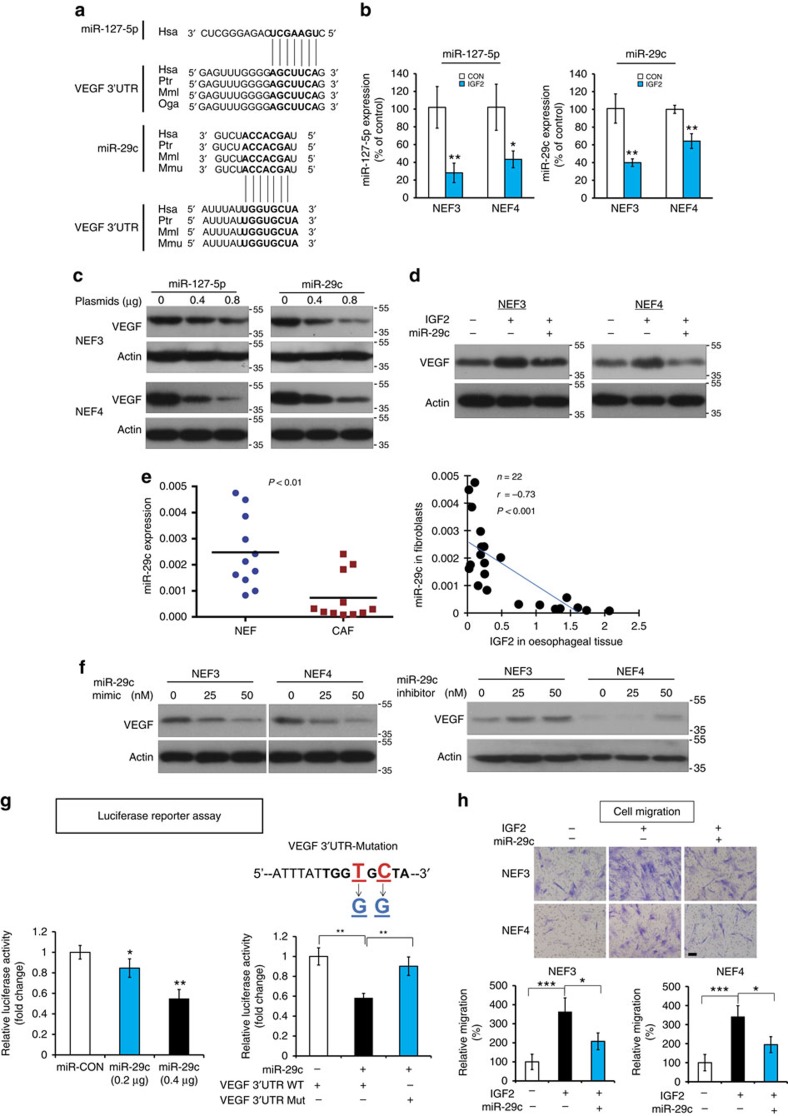
miR-29c mediates the regulation of IGF2 on VEGF in fibroblasts. (**a**) Base pairing between 3′UTR of VEGF and miR-127-5p and miR-29c, respectively. (**b**) Quantification of miR-127-5p and miR-29c expressions in IGF2-treated fibroblasts by TagMan miRNA assay. (**c**) Western blot analysis showing the expression of VEGF in the fibroblasts transfected with miR-127-5p and miR-29c plasmids, respectively. (**d**) VEGF expression was determined in the fibroblasts transfected with miR-29c or miR-CON in the presence or absence of IGF2. (**e**) Comparison of miR-29c expression level between CAFs and matched NEFs from 11 ESCC patients (left panel; paired *t*-test), and correlation with oesophageal tissue IGF2 expression (right panel; Pearson's rank correlation coefficient). (**f**) VEGF expression in the fibroblasts transfected with miR-29c mimic (left panel) or inhibitor (right panel). (**g**) Luciferase activity in fibroblasts co-transfected with miR-29c and wild-type or mutant VEGF 3'UTR luciferase reporter plasmids. (**h**) miR-29c abrogated the stimulatory effect of IGF2 on migration of fibroblasts (scale bar, 100 μm). Three biological replicates were performed for *in vitro* assays. Bars, s.d.; *P<0.05; **P<0.01; ***P<0.001 by Student's *t*-test.

To investigate the mechanisms by which IGF2 regulates VEGF in fibroblasts, we initiated a screening for candidate miRNAs, which can potentially regulate VEGF expression. Three miRNA target prediction software were utilized to search for the miRNAs that may bind to the 3′UTR of VEGF. Among the candidate miRNAs, 40 miRNAs with highest scores (Supplementary Table 3) were selected and real-time RT-PCR was performed to further screen for miRNAs that were downregulated in the fibroblasts after IGF2 treatment. This approach allowed us to narrow the list of candidate miRNAs down to miR-127-5p and miR-29c, both of which satisfied the criteria of having seed regions that perfectly matched with the 3′UTR of VEGF (Fig. 3a) and were downregulated in fibroblasts upon IGF2 treatment (Fig. 3b). Western blot analysis showed that transfection with the plasmids expressing miR-127-5p and miR-29c resulted in reduction of VEGF expression in fibroblasts (Fig. 3c). Interestingly, when we overexpressed these miRNAs in fibroblasts, genetic enforcement of only miR-29c (Fig. 3d), but not miR-127-5p, abrogated the upregulation of VEGF expression induced by IGF2, implying that miR-29c may mediate the effect of IGF2 on VEGF. In the 11 pairs of ESCC and adjacent normal tissues mentioned in the previous section, miR-29c expression was lower in CAFs than in NEFs (Fig. 3e, left panel), and was inversely correlated with IGF2 expression (Fig. 3e, right panel).

Next, gain- and loss-of-function experiments using miR-29c mimic and inhibitor confirmed the regulation of VEGF by miR-29c in fibroblasts (Fig. 3f). In the luciferase reporter assay, overexpression of miR-29c led to a dose-dependent decrease in VEGF 3′UTR reporter expression (Fig. 3g, left panel), but there was no repression of luciferase activity in the fibroblasts co-transfected with miR-29c and mutant VEGF 3′UTR (Fig. 3g, right panel), indicating that miR-29c directly binds to the 3′UTR of VEGF. At the functional level, Boyden chamber assay showed that the enhanced migration of fibroblasts induced by IGF2 could be attenuated by overexpression of miR-29c (Fig. 3h). Taken together, these data suggest that IGF2 induces the activation of fibroblasts to produce VEGF via the mediation of miR-29c.

### Regulation of miR-29c by IGF2 is p53-dependent

**Figure 4:**
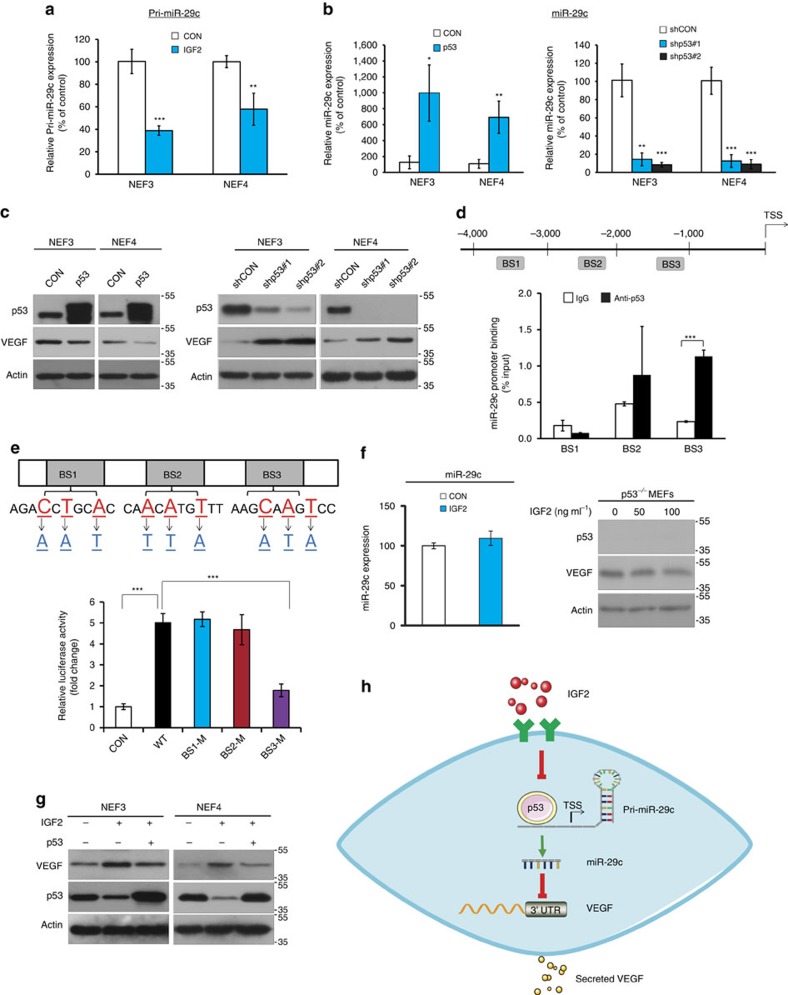
p53-dependent regulation of miR-29c by IGF2. (**a**) Quantification of pri-miR-29c level in IGF2-treated fibroblasts using TagMan pri-miRNA assay. Data were normalized to U6 expression. (**b**,**c**) Fibroblasts were transfected with p53 overexpression or knockdown plasmids, and then expression levels of miR-29c (**b**) and VEGF (**c**) were determined by TagMan miRNA assay and western blot, respectively. (**d**) Three putative p53 binding sites in the promoter of miR-29c were identified by *in silico* prediction, and the enrichment of p53 in miR-29c promoter region was determined by ChIP. (**e**) Diagram illustrating the site-specific mutations introduced in the reporter plasmid for miR-29c promoter (Pgl3-hsa-miR29c-pro-BS3-WT) (upper panel). Lower panel showed the luciferase activity in fibroblasts transfected with p53 and wild type (WT) or mutated (M) miR-29c promoter. (**f**) Tagman miRNA assay and western blot analysis showing the expression of miR-29c and VEGF in p53 null fibroblasts upon IGF2 treatment, respectively. (**g**) VEGF expression in fibroblasts transfected with p53 or vector control in the presence or absence of IGF2. (**h**) Schematic diagram illustrating how IGF2 can induce fibroblasts to secrete VEGF via the mediation of miR-29c in a p53-dependent manner. Three biological replicates were performed for *in vitro* assays. Bars, s.d.; *P<0.05; **P<0.01; ***P<0.001 by Student's *t*-test.

The mechanism by which IGF2 negatively regulates miR-29c expression was investigated. Results from TaqMan pri-miRNA assay showed that the addition of rhIGF2 to fibroblasts decreased the expression of pri-miR-29c (Fig. 4a), the most upstream RNA molecule in miR-29c biogenesis, which suggests that IGF2 regulates miR-29c at transcriptional level. By using *in silico* prediction, three potential p53 binding sites were identified in the promoter region of miR-29c, leading us to speculate that p53 may participate in the regulation of miR-29c by IGF2.

Gain- and loss-of-function experiments demonstrated that p53 had a positive regulatory effect on the expression of miR-29c (Fig. 4b). Western blot analysis showed that p53 negatively regulated the expression of VEGF in fibroblasts (Fig. 4c). Chromatin immunoprecipitation-quantitative PCR assay was performed to determine whether there is physical interaction between p53 protein and the promoter region of miR-29c. Of the three potential p53 binding sites in the promoter region of miR-29c (designated as BS1, BS2, BS3), enrichment of BS3 fragment, but not BS1 or BS2, was observed when cells were co-transfected with plasmids expressing p53 and wild type miR-29c promoter (Fig. 4d). To further confirm the direct binding between p53 and the promoter of miR-29c, site-specific mutations were performed and luciferase assay demonstrated that only BS3, but not the others, functions as the p53-responsive element (Fig. 4e). These results collectively suggest that p53 plays a crucial role in mediating the regulation of IGF2 on the expression of miR-29c by directly binding to the promoter of miR-29c. The significance of p53 in this regulatory mechanism was further tested using p53-null mouse embryonic fibroblasts (p53^−/−^ MEFs). Unlike p53-expressing fibroblasts that showed dose-dependent increase in VEGF in response to IGF2 (Supplementary Fig. 1h), there was no change in miR-29c and VEGF levels in p53^−/−^ MEFs upon IGF2 treatment (Fig. 4f). Moreover, we found that while exogenous IGF2 decreased the expression of p53 in normal oesophageal fibroblasts, the expressions of p-AKT and p-MDM2, which are upstream regulators of p53, were increased (Supplementary Fig. 1h), suggesting that IGF2 may regulate p53 expression through the AKT/MDM2 pathway. Subsequent experiments showed that overexpression of p53 in NEFs abolished the elevation of VEGF induced by IGF2 (Fig. 4g). A schematic diagram depicting the functions of p53 and miR-29c in the regulatory mechanism of IGF2 on VEGF expression in fibroblasts is given in Fig. 4h.

### Id1-expressing tumour instigates VEGFR1^+^ bone marrow cells

**Figure 5:**
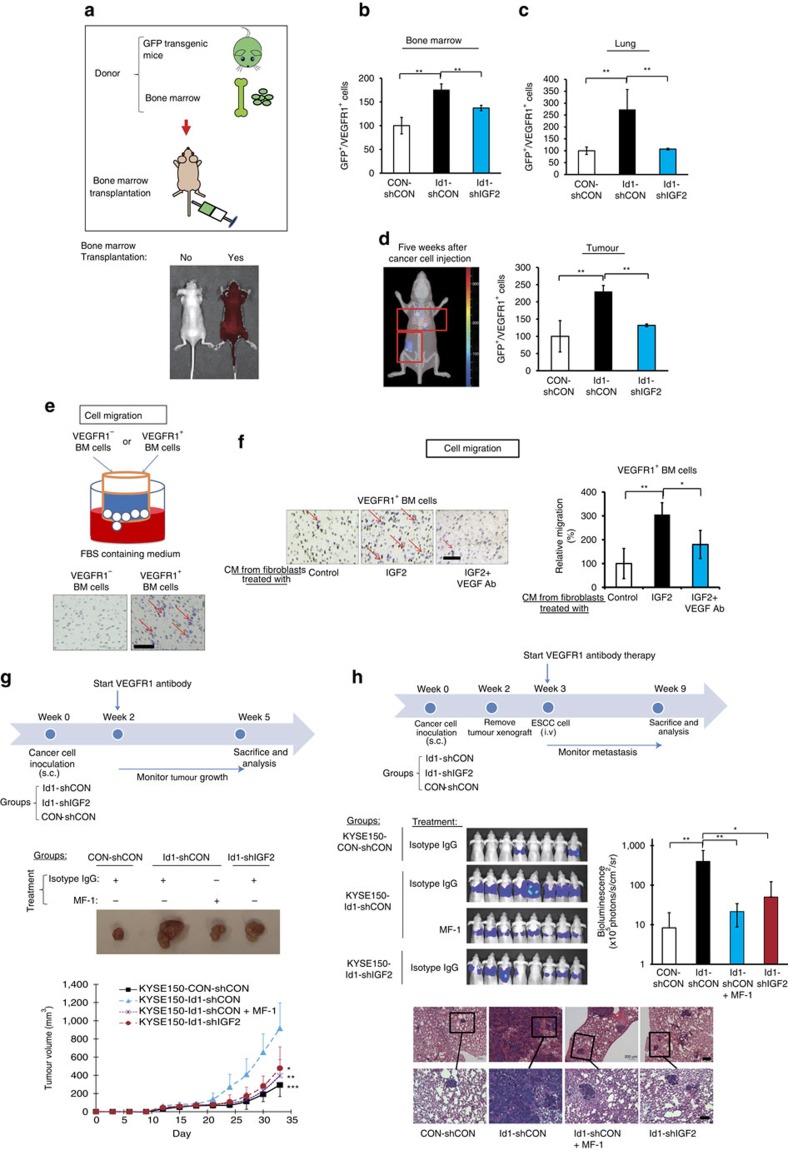
Systemic instigation of VEGFR1^+^ bone marrow cells by Id1-induced IGF2. (**a**) Illustration within the box shows the experimental scheme for bone marrow transplantation; lower panel shows representative fluorescence images of recipient control mice without (labelled ‘No') and with bone marrow transplantation (labelled ‘Yes'); the red intensity indicated strong GFP signals. (**b**,**c**) Quantification of VEGFR1^+^ cells in bone marrow (**b**) and lung (**c**) of mice (female 6–8-week-old nude mice, *n*=3 per group) from each experimental group by flow cytometry analysis. (**d**) Representative three-dimensional tomography image of mice showing accumulation of GFP-positive cells in the thoracic region and in the subcutaneous tumour indicated by red frames (left panel) and quantification of VEGFR1^+^ cells in the subcutaneous tumours (right panel). (**e**) Migrating ability of sorted VEGFR1^+^ and VEGFR1^−^ bone marrow cells compared by FBS-gradient induced cell migration assay (scale bar, 100 μm). (**f**) The migration ability of VEGFR1^+^ bone marrow cells in response to the attraction of conditioned medium from IGF2-induced fibroblasts in the presence or absence of VEGF antibody was compared (scale bar, 100 μm). (**g**) Outline of experimental scheme and the effect of VEGFR1 blockade on tumour growth (female 6–8-week-old nude mice, *n*=6 per group; scale bar, 1 cm). (**h**) Experimental scheme and bioluminescence imaging showing effect of VEGFR1 blockade on lung metastasis; sections of lung tissue (H & E stained) are shown in the bottom panel (female 6–8-week old nude mice, *n*=8 per group; scale bars, 200 μm and 100 μm for top and bottom rows of photomicrographs, respectively). Bars, s.d.; *P<0.05; **P<0.01, ***P<0.001 by Student's *t*-test.

In view of the importance of bone marrow-derived cells (BMDCs) in tumour progression^16^, we investigated whether Id1-expressing tumour can mobilize and home BMDCs to tumour and pre-metastatic sites. To establish a traceable system, we transplanted the bone marrow of transgenic mice expressing green fluorescent protein (GFP) into groups of lethally irradiated recipient nude mice (Fig. 5a). Different groups of recipient mice were inoculated subcutaneously with human ESCC (KYSE150) cells expressing Id1, Id1-shIGF2 and control vectors, respectively, 2 weeks after bone marrow transplantation. To identify and characterize the BMDC subpopulations that were influenced by Id1-overexpressing tumour xenografts, subpopulations of BMDCs that are known to contribute to tumorigenesis^5^, identified by different cell-surface antigens including CD11b, F4/80, CD335, Gr-1, Tie-2, VEGFR1 and VEGFR2, were analysed. We found an enrichment of GFP^+^/VEGFR1^+^ cells (Fig. 5b and Supplementary Fig. 5a), but not the other subpopulations (Supplementary Fig. 5b), in the bone marrow of mice bearing Id1-expressing tumours, compared with the Id1-shIGF2 and the vector control groups. Flow cytometry analysis also showed similar enrichment of GFP^+^/VEGFR1^+^ bone marrow cells in the lungs (which is a common site of ESCC metastasis) of the Id1 group (Fig. 5c and Supplementary Fig. 5a). Five weeks after implantation of cancer cells, an accumulation of GFP^+^ cells was detected in the tumour xenografts and thoracic region using three-dimensional *in vivo* mouse imaging (Fig. 5d, left panel). Flow cytometry analysis of the tumour xenografts revealed an increase in GFP^+^/VEGFR1^+^ cells, but not the other subpopulations, in the Id1 group only (Fig. 5d, right panel and Supplementary Fig. 5a). These results not only confirmed that VEGFR1^+^ BMDCs were recruited to the ‘primary' tumour and secondary sites, but also suggested that Id1-induced IGF2 plays an important role in activating and mobilizing VEGFR1^+^ BMDCs.

Bone marrow cells collected from nude mice without prior treatment were sorted into VEGFR1^+^ and VEGFR1^−^ populations (Supplementary Fig. 6a). *In vitro* experiments showed that VEGFR1^+^ bone marrow cells exhibited higher migratory potential than VEGFR1^−^ bone marrow cells (Fig. 5e). Furthermore, exposure to conditioned medium from IGF2-activated fibroblasts could enhance the migratory potential and proliferation of VEGFR1^+^ bone marrow cells, whereas the presence of VEGF antibody attenuated such effects (Fig. 5f and Supplementary Fig. 6b). The presence of VEGF antibody could also abolish the enhanced migration of VEGFR1^+^ bone marrow cells attracted by the co-culture of fibroblasts with Id1-expressing cancer cells (Supplementary Fig. 6c). The preponderance of VEGFR1^+^ BMDCs in the growing tumours and the lungs of the Id1 group prompted us to explore whether inhibiting the activity of these cells by VEGFR1 inactivation could suppress tumour growth and metastasis. Using the *in vivo* models described in the previous paragraph, we found that VEGFR1 inactivation by treatment with anti-mouse VEGFR1-specific neutralizing antibody (MF-1) was as effective as IGF2-knockdown in retarding the rapid growth of Id1-shCON tumour (Fig. 5g) and reducing the percentage of VEGFR1^+^ cells in tumour xenografts (Supplementary Fig. 7). To determine if other VEGFR1^+^ BMDC-enriched sites in the tumour macroenvironment (such as the lungs) favoured metastasis, the subcutaneous tumours were allowed to grow *in vivo* for 2 weeks to prime the tumour macroenvironment. The tumours were then removed and Luc-expressing KYSE150 cells were introduced intravenously into the circulation for experimental metastasis assay. Bioluminescent imaging showed that lung metastasis occurred most rapidly in the mice bearing Id1-expressing tumour, and that treatment with MF-1 to inactivate host VEGFR1 substantially reduced lung metastasis (Fig. 5h).

Taken together, these data showed that Id1-induced IGF2 from primary tumours can affect the tumour micro- and macroenvironment by systemically activating and mobilizing VEGFR1^+^ BMDCs to facilitate tumour growth and metastasis.

### Fibroblast-derived VEGF systemically enriches VEGFR1^+^ cells

**Figure 6:**
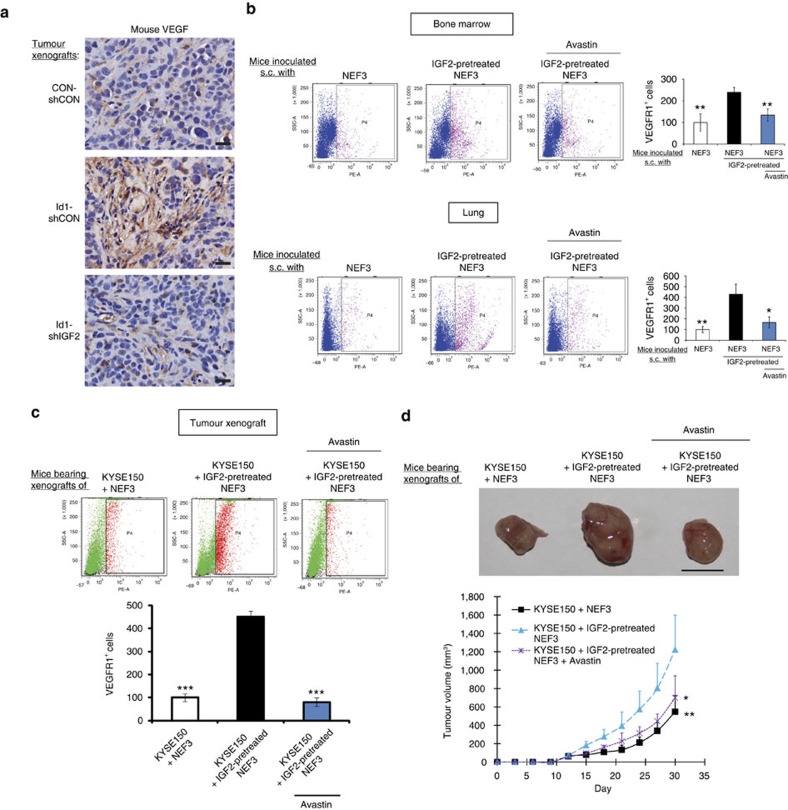
Fibroblast-derived VEGF enriches VEGFR1^+^ cells bone marrow and pre-metastatic sites. (**a**) Immunohistochemical expression of mouse VEGF in the tumour xenografts established with KYSE150-Id1, KYSE150-Id1-shIGF2 or vector control cells (scale bar, 20 μm). (**b**) Flow cytometry data showing the expression of VEGFR1^+^ cells in bone marrow (upper panel) and lungs (lower panel) of mice with subcutaneous implantation of indicated fibroblasts and treatment (female 6–8-week-old nude mice, *n*=3 per group). (**c**,**d**) Flow cytometry analysis of VEGFR1^+^ cells (**c**) and comparison of tumour volume (**d**) of subcutaneous tumour xenografts established from co-implantation of KYSE150 cells and indicated NEFs in the presence or absence of Avastin treatment. Photographs show representative tumours of the three groups (female 6–8-week-old nude mice, *n*=6 per group; scale bar, 1 cm). Bars, s.d.; *P<0.05; **P<0.01; ***P<0.001 by Student's *t*-test.

The data in Fig. 1b showed that nude mice bearing Id1-overexpressing ESCC tumours had elevated serum VEGF that was host-derived. To determine if this was attributed to increased VEGF expression in IGF2-activated stromal fibroblasts, sections of tumour xenografts expressing Id1-overexpressing, Id1-shIGF2, or vector control were immunostained for mouse VEGF expression. The results showed obvious increase of VEGF immunostaining in the stroma of Id1-overexpressing tumours (Fig. 6a). To confirm that fibroblast-derived VEGF can instigate VEGFR1^+^ bone marrow cells, NEFs or IGF2 pre-treated human NEFs were injected subcutaneously into the flanks of mice, with a sub-group of mice treated with anti-human VEGF antibody (Avastin). As showed in Fig. 6b, after 1 week, there were more VEGFR1^+^ cells in the bone marrow of mice inoculated with IGF2-pretreated fibroblasts compared with the control group, and this effect was abolished by Avastin treatment. Because similar changes were detected in VEGFR1^+^ cell population in the lungs (Fig. 6b), our results also established that activated fibroblasts can exert their influence remotely on pre-metastatic niches by endocrine instigation via fibroblast-secreted VEGF. To investigate whether fibroblast-derived VEGF could recruit VEGFR1^+^ cells to growing tumours, the experiment was repeated using NEFs or IGF2-pretreated NEFs mixed with ESCC cells (KYSE150) for establishment of subcutaneous tumour xenografts. After 5 weeks, we found robust increase in VEGFR1^+^ cells in the latter group, whereas treatment with Avastin significantly attenuated this effect. In addition, comparison of tumour size showed that presence of IGF2-pre-treated fibroblasts could drive tumour growth, and that Avastin treatment could abolish this effect (Fig. 6c,d). All these data provide solid evidence to support that stromal VEGF from fibroblasts drives VEGFR1^+^ bone marrow cells. Importantly, our experimental models showed that specific blockade of fibroblast-derived VEGF using Avastin was sufficient to suppress oesophageal tumour growth.

### Id1-expressing tumour-primed bone marrow promotes metastasis

**Figure 7:**
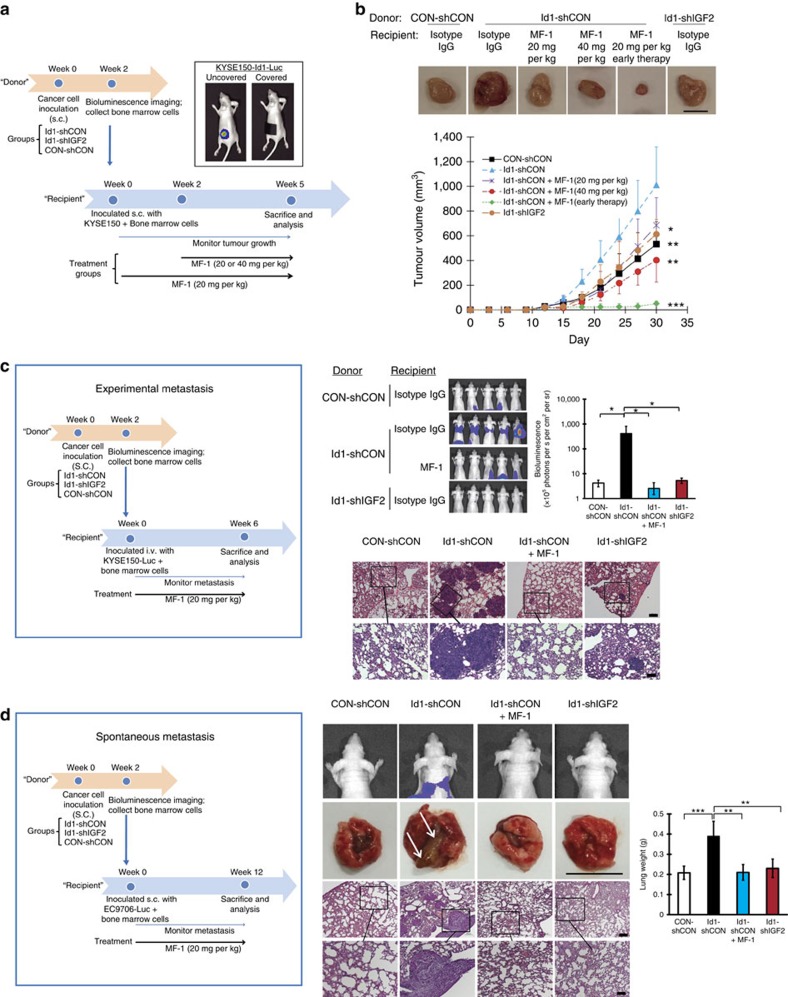
VEGFR1^+^ bone marrow cells interact with ESCC cells to promote tumour growth and metastasis. (**a**) Experimental scheme of bone marrow admixture-tumour xenograft model. Bioluminescence imaging (inset) showed absence of spontaneous metastasis in the lungs 2 weeks after the mice were subcutaneously injected with Luc-expressing KYSE150-Id1 cancer cells. (**b**) Representative photos and growth curves of tumours among the experimental groups (female 6–8-week-old nude mice, *n*=6 per group; scale bar, 1 cm). (**c**) Experimental scheme of the bone marrow admixture-experimental metastasis model (left panel); bioluminescence imaging and quantification of lung metastasis (right panel) (female 6–8-week-old nude mice, *n*=5 per group; scale bars, 200 μm and 100 μm for top and bottom rows of photomicrographs, respectively). (**d**) Experimental scheme of the bone marrow admixture-spontaneous metastasis model (left panel); bioluminescence imaging, macroscopic (arrows indicate visible metastases; scale bar, 1 cm) and microscopic pictures of the dissected lungs and quantification of lung weight (right panel) (female 6–8-week-old nude mice, *n*=5 per group; scale bars, 200 μm and 100 μm for top and bottom rows of photomicrographs, respectively). Bars, s.d.; *P<0.05; **P<0.01; ***P<0.001 by Student's *t*-test.

To determine if bone marrow cells primed by the presence of Id1-expressing tumour can directly facilitate tumour growth by interacting with cancer cells, bone marrow was collected 2 weeks after nude mice had been subcutaneously inoculated with KYSE150 cells expressing Id1, Id1-shIGF2 or vector control, and then admixed with parental KYSE150 cells for establishment of subcutaneous tumours in new groups of nude mice (Fig. 7a). This time point was selected because data from Fig. 5b showed that the 2-week duration was sufficient for the Id1-expressing tumours to condition the bone marrow and enrich it with VEGFR1^+^ cells, and yet the tumours of the three different groups were still of comparable volume (Fig. 5g) so the tumour size of the ‘donor' mice would not be a confounding variable in the following experiments. The bone marrow from mice bearing Id1-expressing tumour was found to be most potent in enhancing the growth of the new xenografts; early commencement of VEGFR1 blockade using MF-1, albeit the lower dose used, was the most effective in inhibiting tumour growth, resulting in nearly complete suppression (Fig. 7b).

To study whether the bone marrow cells primed by Id1-expressing tumour could stimulate ESCC cells to metastasize, KYSE150-luc cells were mixed with bone marrow cells from the three groups of ‘donor' mice, and then injected intravenously into the tail vein of ‘recipient' mice (Fig. 7c). We observed that lung metastasis developed most rapidly in the group injected with cancer cells mixed with bone marrow donated by mice bearing Id1-overexpressing tumour xenografts, and that selective VEGFR1 blockade using MF-1 suppressed lung metastasis (Fig. 7c). The experiment was repeated using a spontaneous metastasis model. The results showed that bone marrow cells primed by Id1-overexpressing tumour in ‘donor' mice, when mixed with the EC9706 cells and subcutaneously injected into the flank of nude mice, had the highest propensity to induce spontaneous metastasis (two out of five mice had spontaneous metastasis in the lungs) compared with the control group (none of five mice had spontaneous metastasis in lung) and that MF-1 treatment abolished this effect (no lung metastasis detected in the group) (Fig. 7d). These data collectively suggest that under the instigation of Id1-expressing tumours, the VEGFR1^+^ cells in the bone marrow were endowed with the capability to stimulate ESCC cells to proliferate and metastasize even before they were recruited into primary tumours and secondary sites.

### CXCL5/CXCR2 axis contributes to pre-metastatic niche

**Figure 8:**
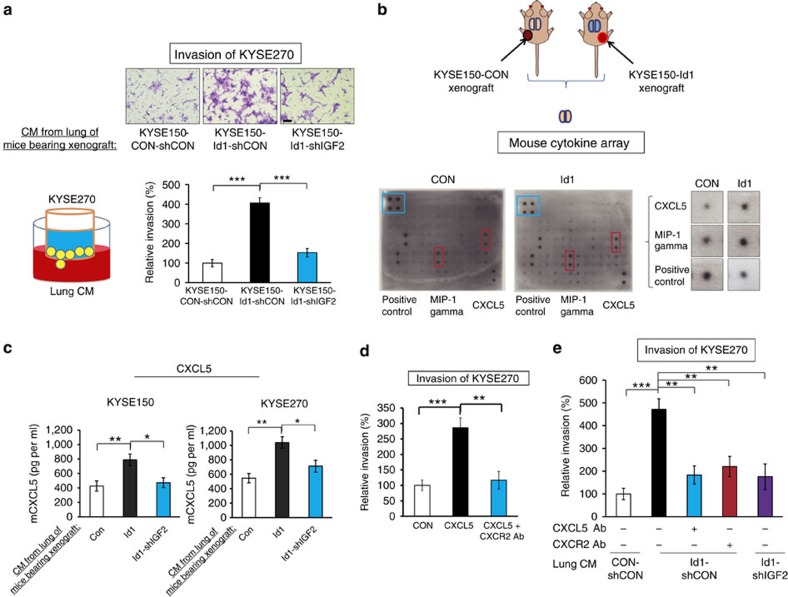
Id1-expressing tumour stimulates the formation of pre-metastatic niches in lungs. (**a**) Invasion ability of ESCC cells induced by the conditioned medium of lung cells from mice bearing the tumours expressing Id1 alone, Id1-shIGF2, or vector control, respectively (scale bar, 100 μm). (**b**) Cytokine profiling of conditioned medium of lung cells from nude mice bearing Id1-expressing and control tumour xenograft, respectively, was performed using a mouse cytokine antibody array. Blue frames indicate the internal positive control and red frames indicate the proteins with markedly increased expression. (**c**) Quantification of CXCL5 level in conditioned medium of lung cells. (**d**) The invasion of KYSE270 cells attracted by CXCL5 in the absence or presence of CXCR2 antibody was determined. (**e**) The invasion ability of KYSE270 cells attracted by conditioned medium from mouse lung cells in the presence or absence of CXCR2 or CXCL5 antibody was compared. Three biological replicates were performed for *in vitro* assays. Bars, s.d.; *P<0.05; **P<0.01, ***P<0.001 by Student's *t*-test.

Different cells in the pre-metastatic niche may collaboratively generate a favourable environment for the invasion and colonization of cancer cells. However, the molecular mechanisms of pre-metastatic niche remain poorly understood. Our results of Boyden Chamber assay showed that the conditioned medium of the lung cell suspension of mice with Id1-overexpressing tumour xenografts was most effective in attracting the invasion of cancer cells (Fig. 8a), suggesting that the lungs of these animals harboured secretory factors that were conducive to colonization of ESCC cells. A mouse cytokine antibody array was used to compare the conditioned medium of lung cell suspension preparations from mice bearing Id1-expressing xenografts and that from control animals to identify the potential players. There was increase in macrophage inflammatory protein-1 gamma (MIP-1γ) and LPS-induced CXC chemokine (also known as LIX, CXCL5 or ENA-78) in the Id1-overexpressing group (Fig. 8b). Since MIP-1γ has no identified human homologue, we focused our attention on CXCL5. Data from ELISA (Fig. 8c) further confirmed the elevation of mouse CXCL5 in the lungs of mice bearing Id1-overexpressing xenografts. Immunohistochemistry showed that CXCL5 was expressed by alveolar epithelial cells (Supplementary Fig. 8). We found that recombinant CXCL5 exerted strong chemotactic effect on invasion of ESCC cells (Fig. 8d). In addition, pre-treatment of lung cell preparations with anti-CXCL5 neutralizing antibody or blockade of its receptor CXCR2 on ESCC cells abrogated the enhanced invasion of ESCC cells attracted by the lung preparations from mice bearing Id1-expressing tumours (Fig. 8e).

## Discussion

**Figure 9:**
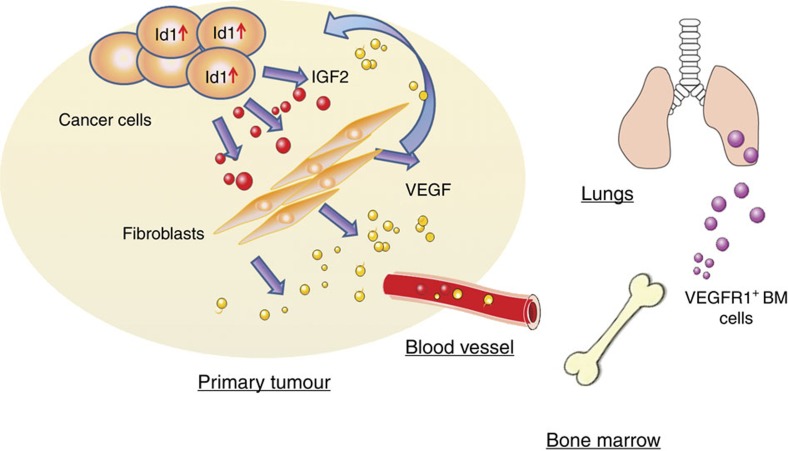
Schematic diagram summarizing the regulatory role of the Id1/IGF2/VEGF/VEGFR1 cascade in oesophageal cancer progression. IGF2 secreted by Id1-expressing cancer cells activates fibroblasts to secrete VEGF which exerts paracrine effects in the tumour microenvironment, as well as instigates VEGFR1^+^ bone marrow cells in the tumour macroenvironment to facilitate distant metastasis.

Id1 is frequently upregulated in a variety of human cancers including ESCC^17^,^18^,^19^. Our previous study showed a positive correlation between Id1 and IGF2 expressions in ESCC^15^. In this study, we showed for the first time that IGF2 secreted by Id1-overexpressing ESCC cells could activate NEFs to secrete VEGF through the mediation of miR-29c in a p53-dependent manner. Our *in vivo* data substantiate that through this mechanism, Id1-expressing ESCC tumours can indirectly promote the functional incorporation of VEGFR1^+^ bone marrow cells into primary tumours and secondary sites to facilitate tumour growth and formation of pre-metastatic niche. Our study demonstrated a novel paradigm of ESCC progression that involves the orchestration of cancer and non-cancer cells in the tumour micro- and macroenvironments by the Id1/IGF2/VEGF/VEGFR1 cascade (Fig. 9).

The roles and mechanisms of fibroblasts in cancer are not fully understood. As increasing evidence revealed that stromal fibroblasts can have opposite influences on tumour development^4^,^20^,^21^, there are still intense debates over whether fibroblasts promote or impede cancer. In addition to stimulating proliferation, migration and tube-formation of endothelial cells (Supplementary Figs 2a–c and 3), the IGF2-activated fibroblasts also promoted tumour malignancy in a multitude of ways such as enhancing the invasion of oesophageal cancer cells (Supplementary Fig. 2d) and migration of bone marrow cells (Fig. 5f) by secreting VEGF. Considering the complexity of the tumour microenvironment, it is likely that other cell types such as macrophages and other immune cells may participate in cancer progression. However, our data clearly demonstrated that fibroblasts are critical players in a network connecting multiple cellular components of the tumour microenvironment that evolve concertedly during cancer progression.

Although the upregulation of VEGF expression by IGF2 had been reported in hepatocellular carcinoma and keratinocytes^22^,^23^, how IGF2 regulates VEGF in a paracrine manner remains largely unknown. We have identified miR-29c as a crucial mediator in the regulation of VEGF by IGF2 (Fig. 3). The miR-29 family is reported to be aberrantly expressed in a variety of human cancers^24^. MiR-29c, located on chr.1q32.2, is highly conserved among mammals, thus suggesting its biological importance^25^. The known target genes of miR-29c, which have cancer-promoting functions, include but are not limited to DNMT3A^26^, ITGB1 (ref. 27), SIRT1 (ref. 28) and cyclin E^29^, some of which have prognostic significance in ESCC. Here, our results showed that p53 regulated miR-29c at transcriptional level by binding to its promoter, and that IGF2 secreted by ESCC cells may downregulate p53 by activating AKT/MDM2, thereby increasing VEGF expression in fibroblasts (Fig. 4). We also identified for the first time the binding site responsible for transcriptional regulation of miR-29c by p53. Because p53 mutation is a highly frequent event in ESCC^30^ whereas fibroblasts are more genetically stable^31^,^32^, this p53-dependent mechanism may explain why cancer cell-secreted IGF2 secreted by ESCC cells exerted paracrine rather than autocrine effect to induce VEGF (Fig. 1b). Taken together, our study has identified a crucial regulatory cascade involving IGF2, p53, miR-29c and VEGF in cancer development.

Oesophageal cancer is the eighth most common cancer worldwide, and patients with distant metastasis have very poor survival rate^33^,^34^. It is now recognized that metastasis is a very inefficient event and only less than 1% of disseminated cancer cells succeed in forming macrometastasis^35^. It was proposed that besides genetic changes in the cancer cells, the condition of the host environment is another equally essential determinant of cancer progress. Since Lyden *et al* demonstrated for the first time that impaired recruitment of bone marrow-derived endothelial and hematopoietic precursor cells can effectively block tumour angiogenesis and tumour growth^36^, other bone marrow cells with tumour-promoting functions, for example CD11b^+^/Gr1^+^ bone marrow-derived myeloid cells, CXCR6-expressing bone marrow mesenchymal stem cells and CD13^+^ myeloid cells, have been identified^37^,^38^,^39^. However, there is also evidence showing that bone marrow cells may have cancer-suppressive activity^40^. In our study, among the various bone marrow cell populations, only VEGFR1^+^ bone marrow cells, which have been defined as hematopoietic progenitor cells that are recruited to distant organs to create a permissive microenvironment that is conducive to cancer metastasis^36^,^41^, were found to be affected by Id1-induced IGF2 and could functionally promote tumour growth and metastasis (Figs 5 and 7). Importantly, our data also showed that VEGFR1 neutralization could suppress tumour growth and metastasis driven by Id1.

Increasing attention is being paid to how primary tumour cells orchestrate the formation of future metastatic sites^42^,^43^, but relatively little is known about what makes the latter receptive to incoming cancer cells. Id1 was identified to be an important mediator of breast cancer metastasis to the lungs^44^,^45^. Interestingly, our own studies with animal models also showed that Id1 increases the potential of intravenously injected ESCC cells to colonize the lungs^14^,^46^. It was thus exciting to discover that in addition to increased recruitment of VEGFR1^+^ BMDCs (Fig. 5c), there was upregulation of CXCL5 in the lungs of nude mice xenografted with Id1-expressing ESCC tumours (Fig. 8b). CXCL5 was first identified as an inflammatory peptide with neutrophil-activating properties^47^. Subsequently, it was demonstrated to broadly participate in various cellular functions associated with cancer development^48^,^49^. The significance of CXCL5 in oesophageal cancer has not been reported. We have provided direct evidence showing that CXCL5 is crucial for the formation of pre-metastatic niches, and that blockade of the CXCL5/CXCR2 axis can inhibit ESCC cell invasion. In the lungs, the cellular source of CXCL5 was likely to be alveolar type II epithelial cells judging from the morphology, location and scattered distribution of CXCL5-immunostained cells, as in the case of lipopolysaccharide-induced inflammation^50^.

In conclusion, we have identified novel elements of a therapeutically targetable signalling cascade and elucidated their underlying molecular mechanisms. A full understanding of the intricacy of cellular interactions and molecular mechanisms in the tumour micro- and macroenvironment will open new rational avenues for therapeutic interventions for cancer patients.

## Methods

### Cell lines

Human ESCC cell lines KYSE150 and KYSE270 obtained from DSMZ (Braunschweig, Germany)^51^, and EC9706 obtained from the Chinese Academy of Sciences (Shanghai, China)^52^, were maintained in RPMI 1640 (Sigma, St Louis, MO, USA) supplemented with 10% fetal bovine serum (FBS) (Invitrogen, Gaithersburg, MD, USA). The KYSE150 and KYSE270 cell lines were used to generate stable Id1-overexpressing and vector control cell lines with or without IGF2 knockdown (designated Id1-shIGF2, Id1-shCON and CON-shCON) using full length Id1 expression plasmid pBabe-puro-Id1 (kindly provided by Professor Joan Massague, Memorial Sloan-Kettering Cancer Center, New York, NY, U.S.A) and pSuper-shIGF2 vector expressing the shRNA against IGF2. A luciferase expression vector generated by subcloning the luciferase coding region into pLenti/V5-D-TOPO (Invitrogen) was used to generate the luciferase-expressing stable cell line KYSE150-Luc and EC9706-Luc cells. The cell lines were authenticated by short tandem repeat profiling, and tested for mycoplasma contamination. The p53 null mouse embryonic fibroblast cell line (p53^−/−^ MEFs), derived from p53^−/−^ mouse embryos, was provided by Professor Randy Y.C. Poon, Hong Kong University of Science and Technology^53^. Fibroblasts were maintained in Dulbecco's Modified Eagle Medium (DMEM) (Sigma) supplemented with 10% FBS. Human umbilical vein endothelial cells obtained from Invitrogen were cultured in M200 medium supplemented with low serum growth supplement (Invitrogen).

### Plasmids

The plasmids expressing p53, that is pcDNA3-flag-p53 (Addgene plasmids 10838; Addgene, Cambridge, MA, USA)^54^ and pLenti6/V5-p53 (Addgene plasmids 22945)^55^ were gifts from Professor Thomas Roberts (Dana-Farber Cancer Institute, Boston, MA, USA) and Professor Bernard Futscher (The University of Arizona, Tucson, AZ, USA), respectively. The p53-knockdown plasmids pLKO-p53-shRNA-427 and pLKO-p53-shRNA-941 (Addgene plasmids 25636 and 25637)^56^ were from Professor Todd Waldman (Lombardi Comprehensive Cancer Center, Washington, DC, USA), with the plasmid expressing scrambled shRNA (Addgene plasmid 1864)^57^ from Professor David Sabatini (Massachusetts Institute of Technology, Cambridge, MA, USA) as control. The BLOCK-iT^TM^ Pol II miR RNAi Expression Vector Kit with EmGFP (Invitrogen) was used to create the vectors expressing miR-29c, miR-127-5p and the scrambled miRNA control (miR-CON). The miR-29c mimics and corresponding negative controls as well as the miRIDIAN anti-miR-29c inhibitor and the negative control were purchased from Ambion (Austin, TX, USA). Luciferase reporter plasmid psiCHECK-2 containing wild type 3′-UTR sequence of VEGF, designated as psiCHECK-2-VEGF-3′UTR-WT, was kindly provided by Professor Kwanghee Baek (Kyung Hee University, Korea)^58^. The firefly luciferase-coding pGL3-basic plasmid containing the promoter of miR-29c, pGL3-miR29c-pro-WT^59^, was kindly provided by Professor Carlos López-Otín (Universidad de Oviedo, Spain). siRNAs including human Id1-specific siRNA (5′-TAAACGTGCTGCTCTACGA-3′ (ref. 60); human Id2-specific siRNA (5′-CACGGATATCAGCATCCTG-3′), human Id3-specific siRNA (5′-TCCTACAGCGCGTCATCGA-3′)^61^ and human Id4-specific siRNA (5′-AGATCCTGCAGCACGTTATCG-3′)^62^ were used to knockdown Id genes.

### Human tissue and serum samples

Eleven cases of human ESCC and the corresponding adjacent normal oesophageal tissues (resected at least 5 cm away from the tumour lesion) were freshly collected from patients undergoing surgical resection of primary oesophageal tumour at Queen Mary Hospital, Hong Kong from 2011 to 2014. None of these patients had received neo-adjuvant chemotherapy or radiotherapy. Serum samples from 100 ESCC patients (without prior chemo- or radiotherapy) and 50 healthy individuals were obtained from Queen Mary Hospital (Hong Kong) and Shantou University Medical College (Shantou, China). Use of all human samples that were obtained with informed consent from the patients was approved by the committees for ethical review of research involving human subjects at the Queen Mary Hospital and Shantou University.

### Isolation of fibroblasts

Cancer-associated fibroblasts (CAFs) and their paired NEFs were isolated from fresh specimens of primary ESCC tumours and adjacent non-tumour tissues (resected at least 5 cm away from the tumour lesion) that were collected from patients who underwent surgical resection at the Queen Mary Hospital (Hong Kong). Briefly, the tissues were minced into small pieces in sterile phosphate-buffered saline (PBS) solution and cultured in DMEM supplemented with 10% FBS. The tissue fragments were incubated for about 14 days and early-passage (passage 1–3) fibroblasts were cryopreserved for later use. The fibroblasts used in this study had undergone no more than 10 passages. *TP53* mutation status in NEF3 and NEF4 was determined using the primers listed in Supplementary Table 4 (ref. 63) and no *TP53* mutations were detected in these cell lines.

### Drugs and immunoneutralization antibodies

Recombinant human IGF2 and CXCL5 were from PeproTech (Rocky Hill, NJ, USA). The neutralizing antibodies against human IGF2 (#AF-292-NA), VEGF (#AF-293-NA) and CXCR2 (#MAB331-100) were purchased from R&D Company (Minneapolis, MN, USA). Anti-mouse CXCL5 neutralizing antibody (#DS-PB-01247) was from RayBiotech (Norcross, GA, USA). Rat anti-mouse VEGFR1 monoclonal antibody, MF-1, was provided by Eli Lilly and Company (New York, NY, USA). Avastin (Bevacizumab) was purchased from Roche Diagnostics (Mannheim, Germany).

### Collection of conditioned media

After the cancer cells or single-cell suspensions of lungs had been cultured in serum-free medium for 24 h, the conditioned medium was collected and centrifuged at 1,000 *g* for 5 min. For western blot analysis, conditioned medium was concentrated about 40-fold using Centricon Centrifugal filter (Millipore, Billerica, MA, USA).

### Enzyme-linked Immunosorbent Assay (ELISA)

Human VEGF ELISA kit (R&D) and mouse VEGF ELISA kit (RayBiotech) were used to determine the concentration of human and mouse VEGF in the serum of experimental animals or conditioned medium according to the manufacturer's instructions. Human IGF2 ELISA kit (CusaBio, Wuhan, China) was used to determine the expression of IGF2 in ESCC patients and healthy individuals. Mouse CXCL5 ELISA (RayBiotech) was used to analyse CXCL5 expression level in the conditioned medium of lung cell suspensions of nude mice.

### Real-time polymerase chain reaction and TaqMan miRNA assay

Total RNA was isolated using Trizol reagent according to the manufacturer's protocol (Invitrogen). miRNA was converted to cDNA using the miScript II RT Kit (Qiagen, Hilden, Germany), and quantitative PCR (PCR) was performed using the miScript SYBR Green PCR Kit (Qiagen). The specific forward primer sequences for each miRNA are listed in Supplementary Table 3. All the experiments were performed on MyIQTM2 Real-time PCR Detection System (Bio-Rad, Hercules, CA, USA). The expression of miR-29c was also quantified using the TaqMan miRNA Assay Kit (Applied Biosystems, Carlsbad, CA, USA) according to the manufacturer's instructions. Human small nuclear U6 RNA was included as internal control for miRNA detection.

### TaqMan Pri-miRNA assay

TaqMan Pri-miRNA Assay Kit (Applied Biosystems) was used to quantify primary microRNA of miR-29c (pri-miR-29c). Briefly, total RNA was isolated from fibroblast cells with Trizol reagent (Invitrogen). The DNA-free RNA obtained after treating with TURBO DNA-free kit (Ambion, Foster City, CA, USA) was subjected to TaqMan pri-miRNA assay according to the manufacturer's instructions. U6 was used as an internal control.

### Western blot analysis

Cell pellets suspended in lysis buffer (Cell Signaling Technology, Beverly, MA, USA) was centrifuged at 14000 *g* for 30 min at 4 °C. An appropriate amount of protein mixed with protein loading buffer and boiled for 10 min at 95 °C was then loaded into sodium dodecyl sulfate (SDS) polyacrylamide gel for electrophoresis. The gel was transferred to polyvinylidene fluoride membrane. After blocking with 5% fat-free dry milk in Tris-Buffered Saline Tween-20 (TBST) for 1 h at room temperature, the membrane was probed with diluted primary antibody for 1–2 h at room temperature, then washed with TBST and incubated with corresponding horseradish peroxidase-conjugated secondary antibody for 1 h at room temperature. Signals were detected using ECL Plus Western blotting detection system (Amersham, Piscataway, NJ, USA) and observed by BioMax Light Film (Kodak, Rochester, NY, USA). The primary antibodies used included VEGF (#sc-152; 1:500 dilution), Id1 (#sc-488; 1:1000 dilution), Id2 (#sc-489; 1:1000 dilution), Id3 (#sc-490; 1:1000 dilution), Id4 (#sc-13047; 1:1000 dilution), PIGF (#sc-1880; 1:1000 dilution, FGF (sc-79; 1:1000 dilution) and actin (#sc-1616; 1:2000 dilution) from Santa Cruz Biotechnology; phospho-MDM2 (#3521; 1:1000 dilution), phospho-AKT (#9271; 1:500 dilution) AKT (#4691; 1:1000 dilution), p53 (#2524; 1:2000 dilution) from Cell Signaling Technology; IGF2 (#AF-292-NA;1:500 dilution) and CXCL5 (#DS-PB-01247; 1:1000 dilution) from RayBiotech; and α-SMA (#A5228; 1:1000 dilution; Sigma). Full blots of all western blots are presented in Supplementary Figs 9–12.

### Cell proliferation assay

Cell viability was measured using 3-(4, 5-Dimethyl thiazol-2-yl)-2,5-diphenyl tetrazolium bromide (MTT) assay. Briefly, cells were seeded in 96-well plates and incubated with desired medium. At the end of experiment, 20 μl of MTT reagent (5 mg ml^−1^ in PBS) (Sigma) was added and the cells were incubated for 4 h at 37 °C, followed by adding 200 μl DMSO. Absorbance at 570 nm was measured using spectrophotometer.

### *In vitro* cell migration and invasion assay

The motility of cells in response to a chemoattractant was investigated using migration chamber assay (Millipore). The cells of interest were loaded into the upper compartment of the chamber and the chemoattractant as indicated was added to the lower chamber. Following an incubation period of 24 h, the migrated cells were fixed with methanol and stained with crystal violet (0.2%), and then quantified under the microscope in six random fields. BioCoat matrigel invasion chambers (BD Biosciences, San Jose, CA, USA) were used to investigate the invasion of ESCC cells attracted by conditioned medium of fibroblasts. The invaded cells were fixed and stained with crystal violet, and then quantified by submerging the chambers in 1% SDS buffer and measuring the absorbance of the solution at 570 nm.

### Endothelial tube formation assay

Human umbilical vein endothelial cells were seeded in a 96-well plate, which was pre-coated with matrigel (BD Biosciences) and fed with different medium as indicated. After 6 h, capillary-like tubes were imaged and the extent of tube formation was quantified by measuring the total tube length in six random fields from each well using the software Stereo Investigator (MBF Bioscience, VT, USA).

### Immunofluorescence staining

Fibroblasts or cancer cells seeded on the pre-coated coverslips were fixed with 4% paraformaldehyde for 10 min and then permeabilized in Triton X-100 solution (0.1%) at room temperature for 30 min. After blocking with 3% BSA for 30 min, cells were incubated with the primary antibodies against fibronectin (#610077; BD Biosciences) and E-cadherin (#610181; BD Biosciences) at 4 °C overnight. After incubating with fluorochrome-conjugated secondary antibody followed by counterstaining with 4′,6-diamidino-2-phenylindole (DAPI), the slides were mounted in fluorescence mounting medium (DAKO Diagnostics, Mississauga, ON, USA) and the images were captured under a fluorescent microscope.

### Immunohistochemical staining

Paraffin-embedded sections were deparaffinized, rehydrated and then incubated with 0.3% hydrogen peroxide for 30 min. Antigen retrieval was performed in 0.1 M citrate buffer (pH 6.0) for 15 min. After blocking with normal serum followed by incubation with primary antibody, corresponding biotinylated secondary antibody was applied. Peroxidase-conjugated avidin-biotin complex and 3, 3′-diaminobenzidine (DAKO) were used as chromogen, and the sections were counterstained with hematoxylin and eosin (H & E). Microvessel density was calculated as the mean number of CD31-positive vessels in six random fields from representative tumour sections. The CD31 primary antibody (#sc-1506; 1:100 dilution) was purchased from Santa Cruz Biotechnology (Santa Cruz, CA, USA). The CXCL5 (#orb13450; 1:100 dilution) and anti-mouse VEGF antibody (#orb303953; 1:50 dilution) was purchased from Biorbyt Ltd. (Cambridge, Cambridgeshire, UK).

### Flow cytometry analysis and cell sorting

Cell suspensions were labelled with anti-mouse phycoerythrin (PE)-conjugated VEGFR1 (#FAB4711P; R&D Systems), anti-mouse PE-F4/80 (#12-4801; eBioscience Inc., CA, USA), anti-mouse PE-VEGFR2 (#12-5821; eBioscience), anti-mouse PE-CD335 (#12-3351; eBioscience), anti-mouse PE-CD202b (TIE2) (#12-3351; eBioscience), anti-mouse PE-Ly-6G (Gr-1) (#12-5931; eBioscience) and anti-mouse allophycocyanin (APC)-conjugated Cd11b (#53-0112; eBioscience) with corresponding isotype control. Then the samples were analysed on BD FACSCanto II Analyzer (BD Biosciences), and cell sorting was performed on BD FACSAria I Cell Sorter (BD Biosciences). The data analysis was carried out by using FlowJo software (Tree Star Inc., Ashland, OR, USA).

### Prediction of miRNA binding sites on 3′UTR of VEGF

Three software programs including TargetScan (http://www.targetscan.org/vert_50/)^64^, miRanda (http://www.microrna.org/microrna/getExpr Form.do)^65^ and PicTar (http://pictar.mdc-berlin.de/cgi-bin/PicTar_vertebrate.cgi)^66^ were used to predict the putative binding sites on the 3′UTR of VEGF for miRNAs.

### Site-directed mutagenesis and luciferase assay

QuikChange Lightning Site-directed Mutagenesis Kit (Agilent Technologies, Santa Clara, CA, USA) was used to generate mutant constructs for psiCHECK-2-VEGF-3′UTR and pGL3-miR29c-pro. The primers used for generating psiCHECK-2-VEGF-3′UTR-mut were: forward (5′-ttttaatatttgttatcatttatttattgggggtactgtttatccgtaataattgtggggaaa-3′) and reverse (5′-tttccccacaattattacggataaacagtacccccaataaataaatgataacaaatattaaaa-3′). The primers used for generating pGL3-miR29c-pro-mut are listed in Supplementary Table 5. Luciferase activity was measured by using Dual-Luciferase reporter assay according to the manufacturer's instructions (Promega, Madison, WI, USA).

### Chromatin immunoprecipitation-quantitative PCR

Two transcription factor binding site prediction software programs ConTra V2 (ref. 67) and TRRD^68^ were used to identify the potential p53 binding sites in the promoter region of miR-29c. The chromatin immunoprecipitation (ChIP) assay was performed by using simple CHIP enzymatic chromatin IP kit (Cell Signaling, Beverly, MA, USA) according to the manufacturer's manual. In brief, the *in vivo* protein and DNA crosslinking was performed using 37% formaldehyde, followed by sonication and chromatin digestion. The protein–DNA complexes were immunoprecipitated using p53 antibody or negative control IgG antibody. After elution and reversal of crosslinking with proteinase K, the purified DNA was subjected to SYBR Green PCR analysis (Bio-Rad). Relative mRNA expression was calculated using the comparative Ct method after normalization to GAPDH control. The primers used in this protocol were listed in Supplementary Table 6.

### Preparation of mouse bone marrow and tissue suspensions

All animal care and experimental procedures described in this paragraph and subsequent paragraphs were approved by the Committee on the Use of Live Animals in Teaching and Research, University of Hong Kong, or Jinan University. All animals were sex- and age-matched in the animal experiments, and littermates were used. The investigators were not blinded to the experimental groups. Bone marrow was collected by flushing the tibias and femurs with sterile DMEM: Nutrient Mixture F-12 (DMEM/F-12) (Invitrogen). The mixture was passed through an 18-gauge needle and filtered through 70-μm nylon mesh cell strainer to dissociate the cells. The tumour xenografts and lungs harvested from mice were minced to 1 mm^3^ size and digested in collagenase (100 μg ml^−1^) for 20 min at 37 °C with continuous shaking. The sorted VEGFR1^+^ bone marrow cells and lung cells were *ex vivo* expanded in DMEM/F12 medium.

### Bone marrow transplantation

Nude mice (recipient mice) were placed on acidified drinking water (pH 2.5–3.0) for 2 weeks before irradiation. They were exposed to 600 rads of gamma irradiation from Caesium-137 source (MDS Nordion Gammacell 3000 Elan II, Best Theratronics Ltd, Ottawa, Ontario, Canada). Eight to ten hours later, about 2 × 10^6^ bone marrow cells harvested from GFP-expressing donor mice (C57/FVB-GFP) and re-suspended in 100 μl sterile PBS were injected into the tail vein of the recipient mice. Successful engraftment of bone marrow was verified by GFP fluorescence detection using Maestro 2 *in vivo* imaging system (Cambridge Research & Instrumentation, Inc. Woburn, MA, USA).

### Tumour xenograft experiment

In brief, 1 × 10^6^ KYSE150 cells alone or 5 × 10^5^ KYSE150 cells admixed with 5 × 10^5^ bone marrow cells were re-suspended in equal volumes of PBS and matrigel (total volume of 100 μl) and subcutaneously injected into the flanks of mice to establish tumour xenografts. Sub-groups of mice were treated with indicated doses of MF-1 intraperitoneally thrice weekly. For xenografting of fibroblasts alone, 1 × 10^6^ normal oesophageal fibroblasts (NEF3) or IGF2-pretreated NEF3 suspended in equal volumes of PBS and matrigel were subcutaneously injected into the flanks of mice. In another experiment, 1 × 10^6^ NEF3 cells or IGF2 pre-treated NEF3 were admixed with 1 × 10^6^ KYSE150 cells to establish subcutaneous tumour xenografts. Sub-groups of mice were treated with Avastin (5 mg kg^−1^) intraperitoneally thrice weekly when the tumour xenografts reached 5 mm in diameter. During the experiment, the tumour volume was measured every 3 days. Tumour volumes were calculated with the equation *V*=(length × width^2^)/2.

### Experimental metastasis model

Briefly, 1 × 10^6^ luciferase-expressing KYSE150-luc cells or 5 × 10^5^ KYSE150-luc cells admixed with 5 × 10^5^ bone marrow cells were injected intravenously into the animals via the tail vein. Sub-groups of mice were treated with indicated doses of MF-1 intraperitoneally thrice weekly. Isotype IgG was given to the control group. Metastasis was monitored weekly by bioluminescent imaging (Xenogen IVIS 100 *in vivo* imaging system, PerkinElmer, Hopkinton, MA, USA).

### Spontaneous metastasis model

The ESCC cell line EC9706, which is capable of forming spontaneous lung metastasis after subcutaneous implantation in nude mice^69^, was used to generate primary tumours. Briefly, 2 × 10^6^ EC9706-luc cells admixed with 2 × 10^6^ bone marrow cells were inoculated subcutaneously into the flank of nude mice. Sub-groups of mice were treated with indicated doses of MF-1 intraperitoneally thrice weekly with isotype IgG as control. Metastasis was monitored by bioluminescent imaging. At the end of the experiment, the lungs were dissected, weighed and examined histologically for presence of metastases.

### Mouse cytokine antibody array

Single-cell suspensions of lung were cultured in serum-free medium for 24 h and then the culture medium was collected. A mouse Cytokine Antibody Array containing 62 mouse cytokines (#AAM-CYT-3; RayBiotech) was utilized to detect cytokines that were differentially secreted in the lungs of mice bearing Id1-expressing tumour xenografts.

### Gene expression and survival data from patient datasets

Gene expression datasets from patients with breast cancer (GSE 9014) was downloaded from the National Center for Biotechnology Information Gene Expression Omnibus (GEO). R scripting was used to extract the expression values of genes of interests and clinical data from the data matrices. Gene expressions were further divided into high and low levels using median expression level as the cut-off value for Kaplan–Meier survival analyses.

### Statistical analysis

All *in vitro* experiments and assays were repeated at least three times. The data were expressed as mean±s.d. and compared by Student's *t*-test or ANOVA. Animal sample size for each study was chosen on the basis of literature documentation of similar well-characterized experiments, and no statistical method was used to predetermine sample size. Survival analysis was performed by Kaplan–Meier method with the log-rank test, using the Statistical Package for the Social Sciences (SPSS) (SPSS Inc, Chicago, IL). The expression level of miR-29c in NEF and matched CAF was compared using paired *t*-test. The expression levels of serum IGF2 and serum VEGF were compared using unpaired *t*-test. Correlations between Id1 and VEGF, VEGF and IGF2, α-SMA and IGF2, miR-29c and IGF2 were assessed using Pearson's rank correlation coefficient (two-tailed). Univariate and multivariate survival analyses were performed using the Cox proportional hazard model with a forward stepwise procedure (the entry and removal probabilities were 0.05 and 0.10, respectively). *P* values<0.05 were considered as significant for all experiments.

### Data availability

The gene expression datasets from patients with breast cancer referenced in this study (GSE 9014) were downloaded from the National Center for Biotechnology Information Gene Expression Omnibus (GEO). All other data are available within this manuscript and its supplementary information or from the corresponding author upon reasonable request.

## Additional information

**How to cite this article:** Xu, W. W. *et al*. Cancer cell-secreted IGF2 instigates fibroblasts and bone marrow-derived vascular progenitor cells to promote cancer progression. *Nat. Commun.* **8,** 14399 doi: 10.1038/ncomms14399 (2017).

**Publisher's note:** Springer Nature remains neutral with regard to jurisdictional claims in published maps and institutional affiliations.

## Supplementary Material

Supplementary Information Supplementary Figures and Supplementary Tables
