# Supplementary material for: Cancer cell-secreted IGF2 instigates fibroblasts and bone marrow-derived vascular progenitor cells to promote cancer progression
Source: Nat Commun. 2017 Feb 10;8:14399. doi: 10.1038/ncomms14399 (PMC5309924; doi:10.1038/ncomms14399)
Supplement: Supplementary Information — Supplementary Figures and Supplementary Tables [file ncomms14399-s1.pdf]

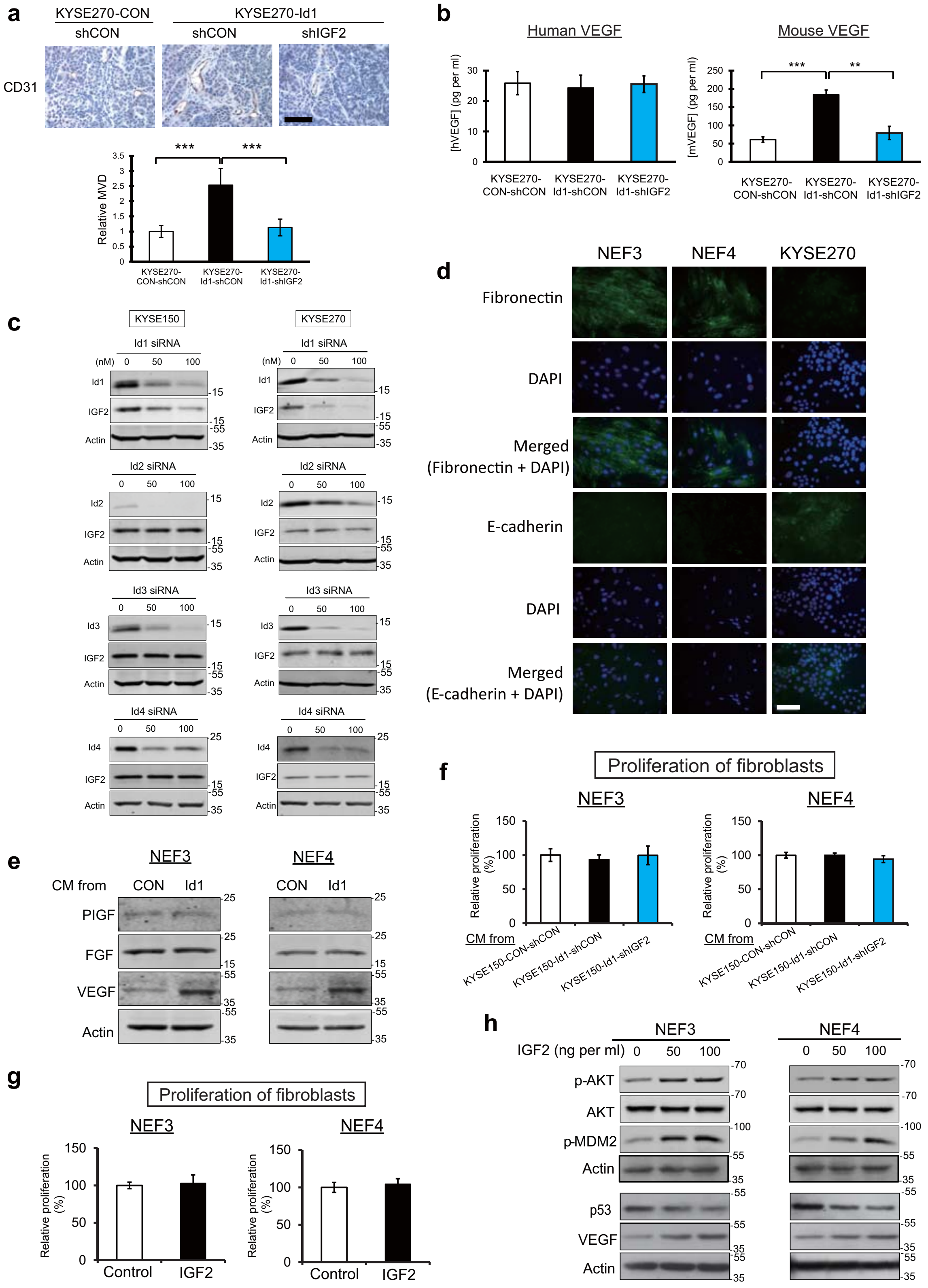

Supplementary Figure 1. Id1-induced IGF2 secreted from ESCC cells increases VEGF-mediated tumor angiogenesis but had no effect on fibroblast proliferation. (**a**) Quantification of CD31-MVD in the tumors established with KYSE270-Id1, KYSE270-Id1-shIGF2 or vector control (female 6-8 week old nude mice, n = 3 per group; scale bar, 100 µm). (**b**) Human and mouse VEGF concentrations were determined in the serum of mice bearing xenografts expressing KYS270-Id1-shCON, KYSE270-Id1-shIGF2 or vector control (female 6-8 week old nude mice, n = 3). (**c**) Western blots showing the effects of Id1, Id2, Id3 and Id4 knockdown on IGF2 by using siRNAs. (**d**) Immunofluorescence showed positive staining for fibronectin but not E-cadherin in fibroblasts compared with ESCC cells (i.e. KYSE270). Cell nuclei were counterstained with DAPI (scale bar, 100 µm). (**e**) Western blot showing the effects of CM from Id1-overexpressing ESCC cells on PIGF, FGF and VEGF. (**f**) MTT assay showed no significant difference in proliferation of fribroblasts fed with the indicated CM from ESCC cells over a 24 hour-period. (**g**) The proliferation of fibroblasts treated with IGF2 (100 ng per ml) for 24 hours was quantified. (**h**) Western blot analysis showing the expression of p-AKT, AKT, p-MDM2, p53 and VEGF in fibroblasts upon IGF2 treatment. Three biological replicates were performed for *in vitro* assays. Bars, s.d.; **, $P < 0.01$; ***, $P < 0.001$ by Student's t-test.

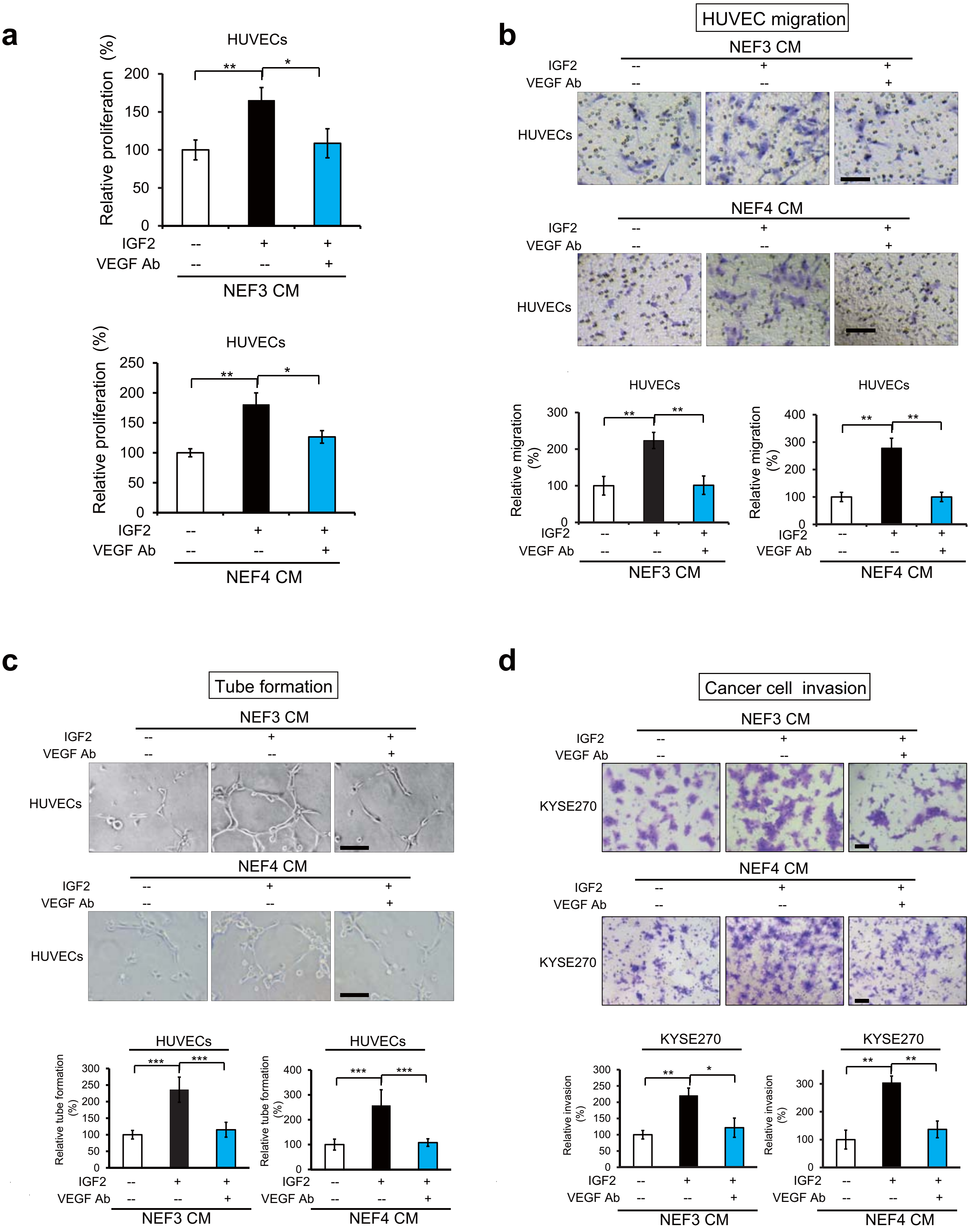

Supplementary Figure 2. Paracrine effects of IGF2-activated fibroblasts on HUVECs and ESCC cells. (**a**) Cell proliferation of HUVECs fed with CM from IGF2-induced fibroblasts in the presence or absence of VEGF antibody was determined by MTT assay. (**b**) Comparison of chemotactic effect of different CM from fibroblasts as indicated on the migration of HUVECs (scale bar, 100 μm). (**c**) Representative images and quantification of tube formation of HUVECs treated with different CM from fibroblasts as indicated (scale bar,100 μm). (**d**) Comparison of invasion of ESCC cells under chemotactic influence of different CM from fibroblasts (scale bar, 100 μm). Three biological replicates were performed for *in vitro* assays. Bars, s.d.; *, $P < 0.05$; **, $P < 0.01$; ***, $P < 0.001$ by Student's *t*-test.

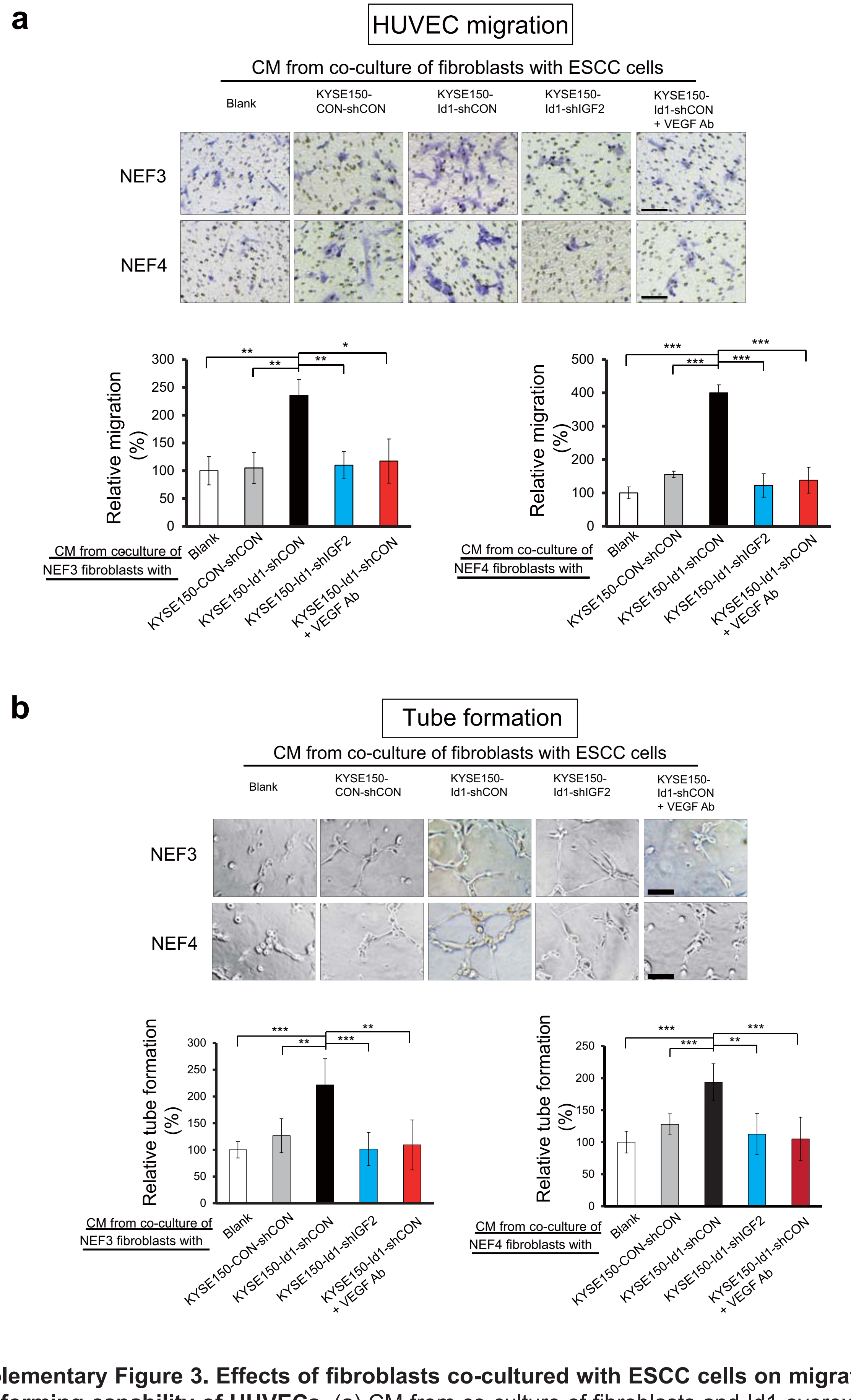

Supplementary Figure 3. Effects of fibroblasts co-cultured with ESCC cells on migration and tube-forming capability of HUVECs. (**a**) CM from co-culture of fibroblasts and Id1-overexpressing ESCC cells had the highest propensity to induce migration of HUVECs, compared with ESCC cells expressing Id1-shIGF2 or vector control (scale bar, 100 μm). (**b**) Treatment with CM from co-culture of fibroblasts and Id1-overexpressing ESCC cells significantly increased the tube-formation activity of HUVECs (scale bar, 100 μm). Three biological replicates were performed for *in vitro* assays. Bars, s.d.; **P*, < 0.05; ***P*, < 0.01; ****P*, < 0.001 by Student's *t*-test.

a

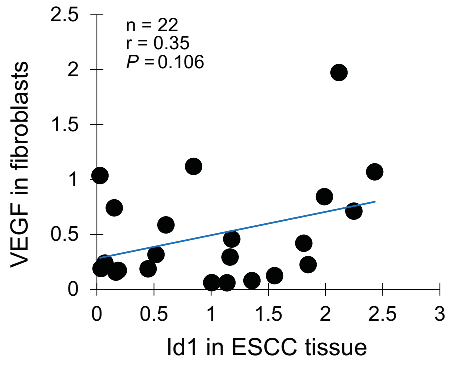

b

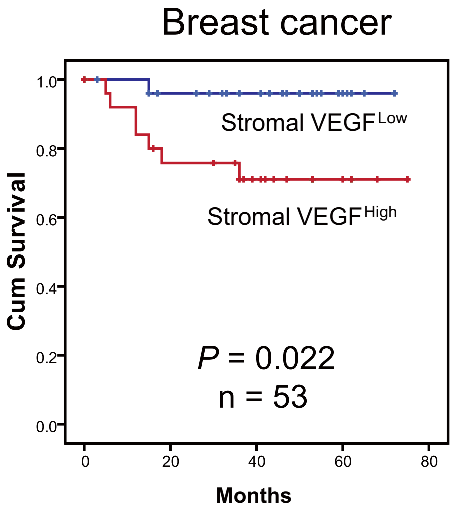

Stromal VEGF	Recurrence		Total
	No	Yes	
Low	25	1	26
High	17	10	27
Total	42	11	53

P = 0.005

Supplementary Figure 4. Correlation analysis of Id1 in ESCC and fibroblast VEGF and clinical relevance of stromal VEGF in breast cancer. (a) Graph correlating expression of Id1 in esophageal tissue with that of VEGF in fibroblasts. Correlation was assessed using Pearson's rank correlation coefficient. **(b)** The association between tumor stromal VEGF expression and the survival rates of patients with breast cancer in GEO database (GSE 9014) was analyzed by Kaplan-Meier analysis; statistical significance was calculated by log-rank test; the Table below showed that the expression of stromal VEGF was significantly correlated with recurrence (*P* = 0.005 by Fisher exact test).

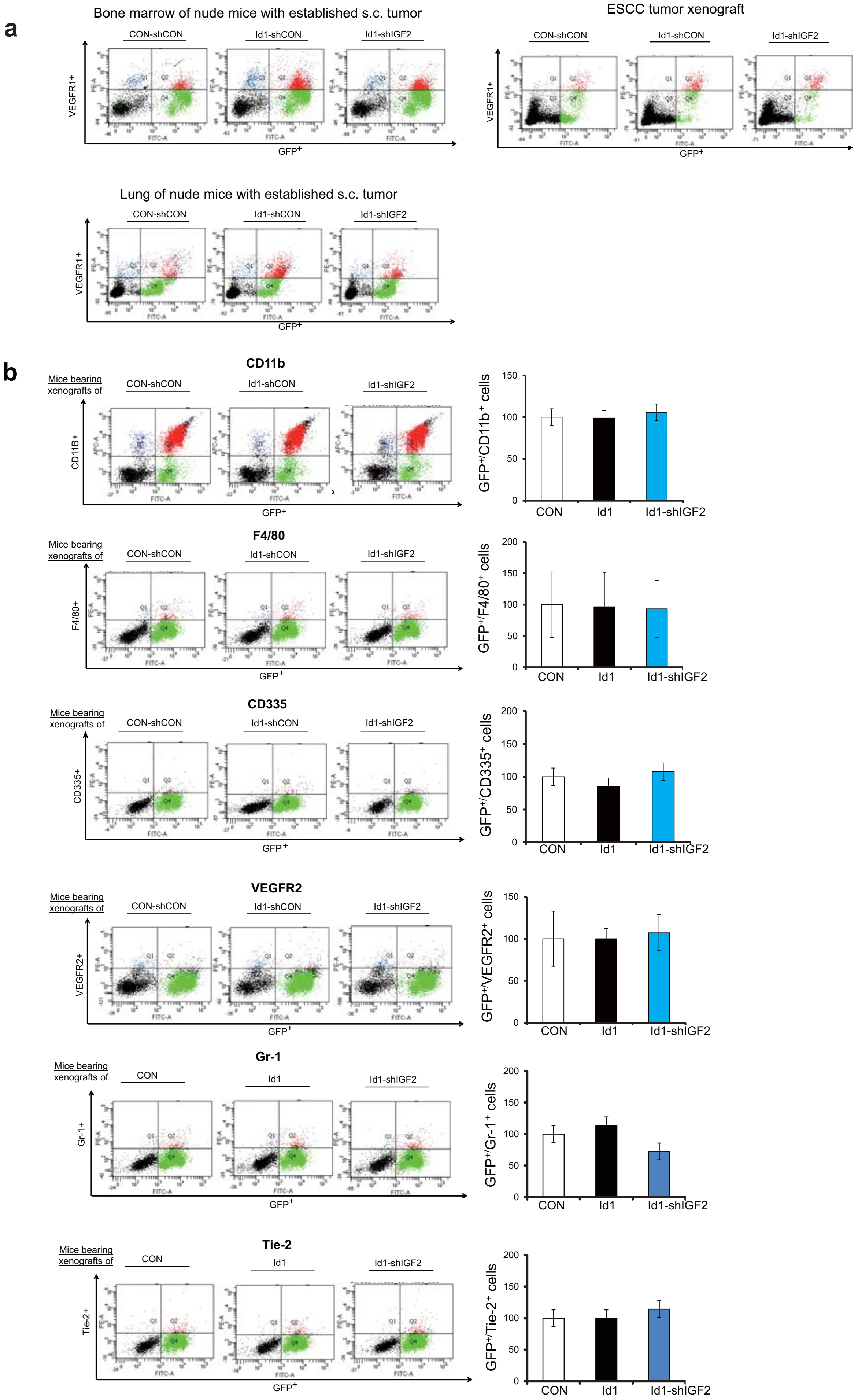

Supplementary Figure 5. Flow cytometric analysis of GFP⁺ cells in bone marrow and lungs of nude mice bearing ESCC tumor xenografts. (**a**) GFP⁺/VEGFR1⁺ cell populations in the bone marrow, lungs and tumor of groups of mice with subcutaneous xenograft of ESCC cells expressing Id1, Id1-shIGF2 or control vectors (female 6-8 week old nude mice, n = 3 per group). (**b**) No significant difference was found in bone marrow CD11b⁺, F4/80⁺, CD335⁺, VEGFR2⁺, Gr-1⁺ and Tie-2⁺ cell populations among the three groups (female 6-8 week old nude mice, n = 3 per group).

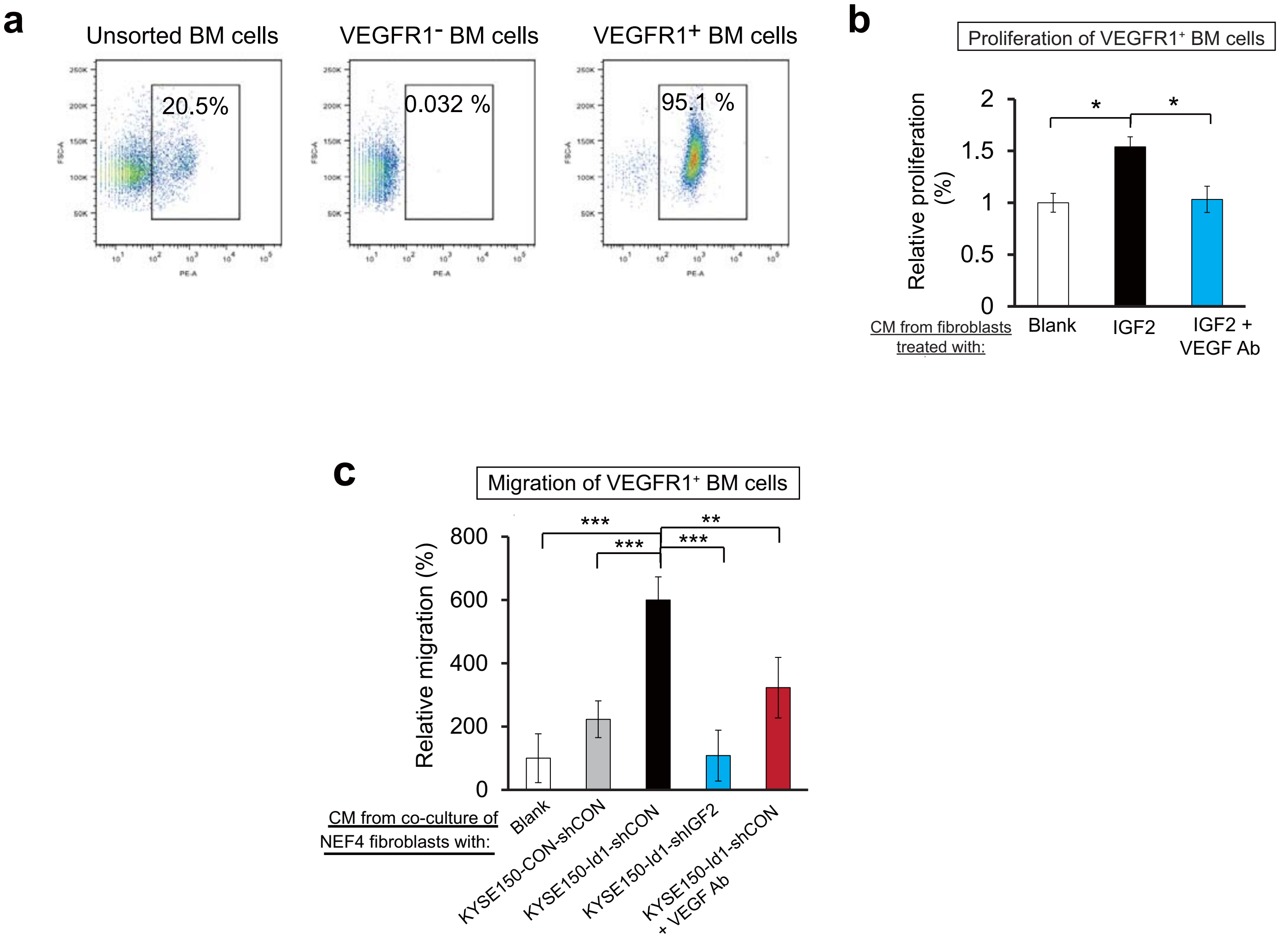

Supplementary Figure 6. Paracrine effects of IGF-activated fibroblasts on migration and proliferation of VEGFR1⁺ bone marrow cells. (**a**) Post-sort analysis of VEGFR1⁺ bone marrow cells. (**b**) Comparison of proliferation of sorted VEGFR1⁺ bone marrow cells treated with the CM from IGF2-induced fibroblasts, in the presence or absence of VEGF antibody. (**c**) The migration of VEGFR1⁺ BMDCs attracted by different CM as indicated was determined. Three biological replicates were performed for *in vitro* assays. Bars, s.d.; *, $P < 0.05$; **, $P < 0.01$; ***, $P < 0.001$ by Student's *t*-test.

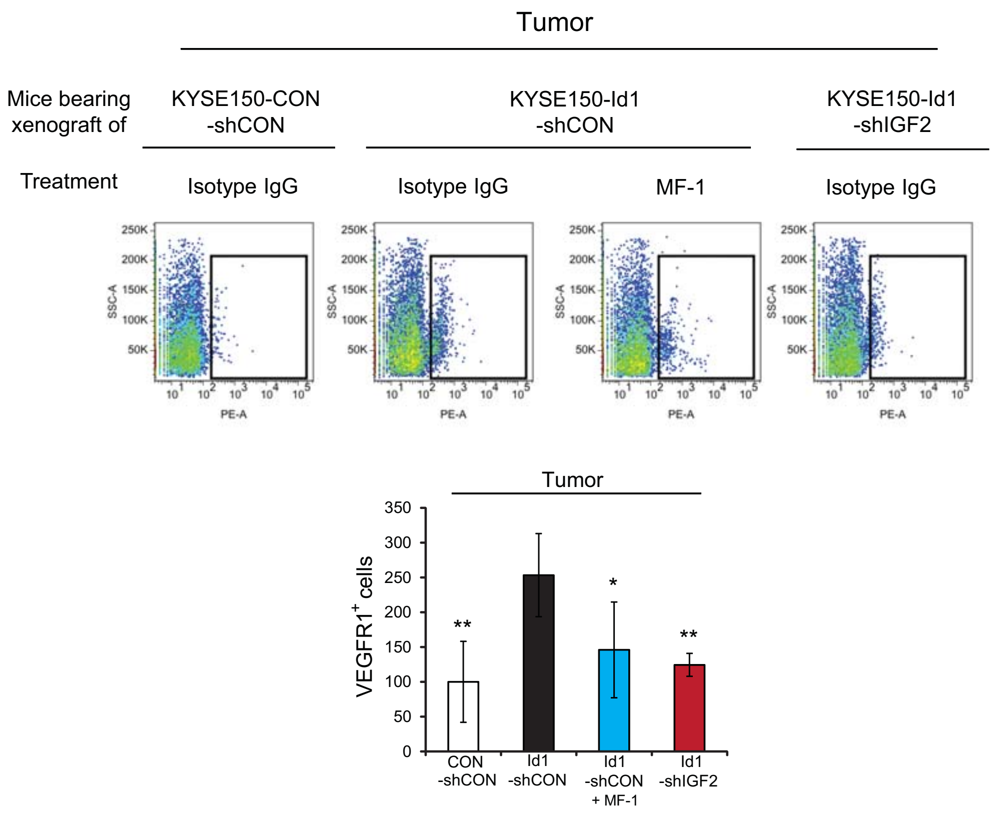

Supplementary Figure 7. Flow cytometric analysis showing the effects of MF-1 treatment and IGF2-knockdown on reducing the expression of VEGFR1$^+$ cells in the tumor of mice bearing Id1-overexpressing tumor xenografts (female 6-8 week old nude mice, n = 3 per group).

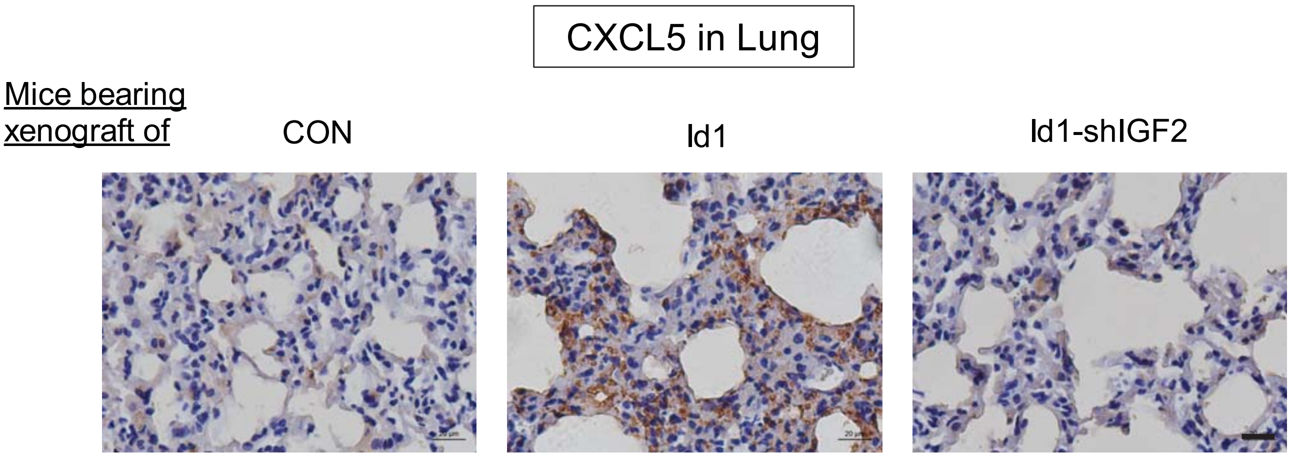

Supplementary Figure 8. Immunohistochemical detection of CXCL5 in the lung tissue of mice bearing indicated tumor xenografts. Scale bar, 20 µm.

Fig. 1c

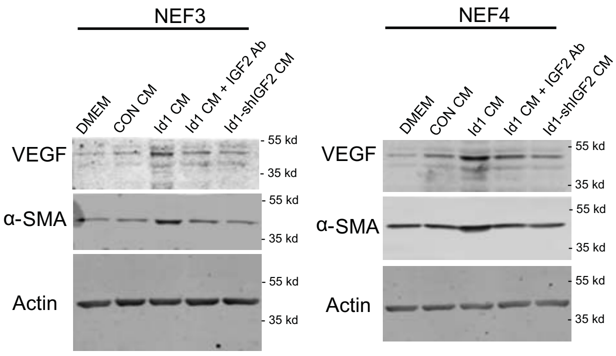

Fig. 1e

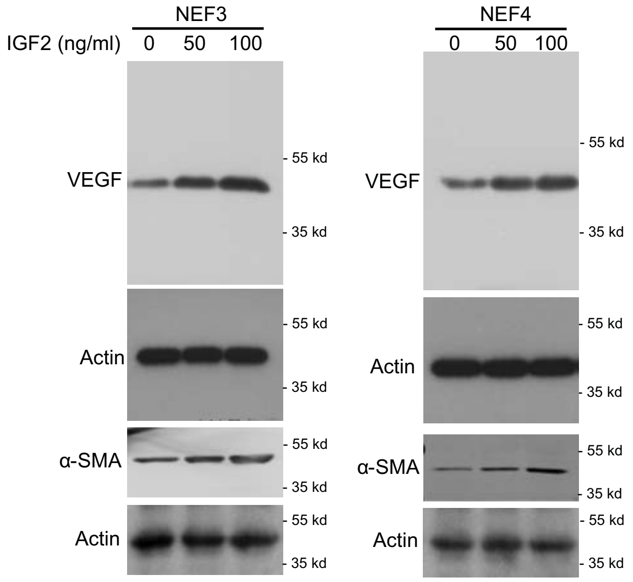

Fig. 2a

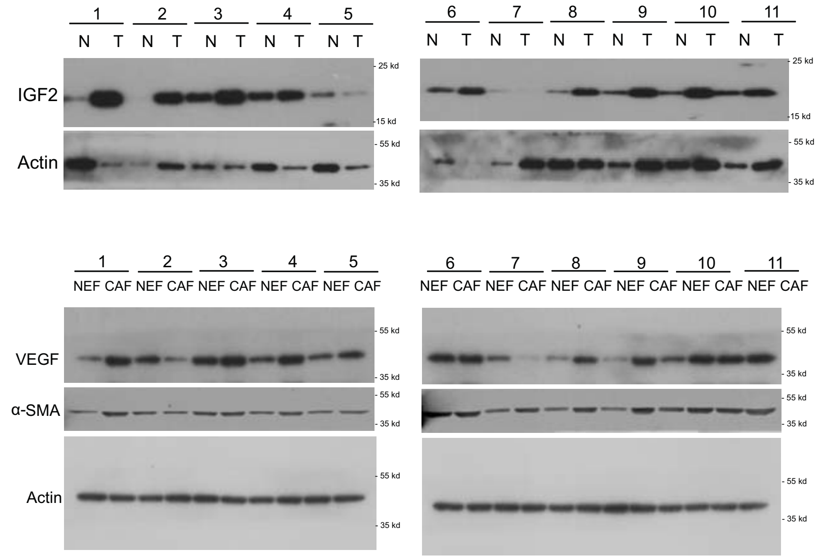

Fig. 3c

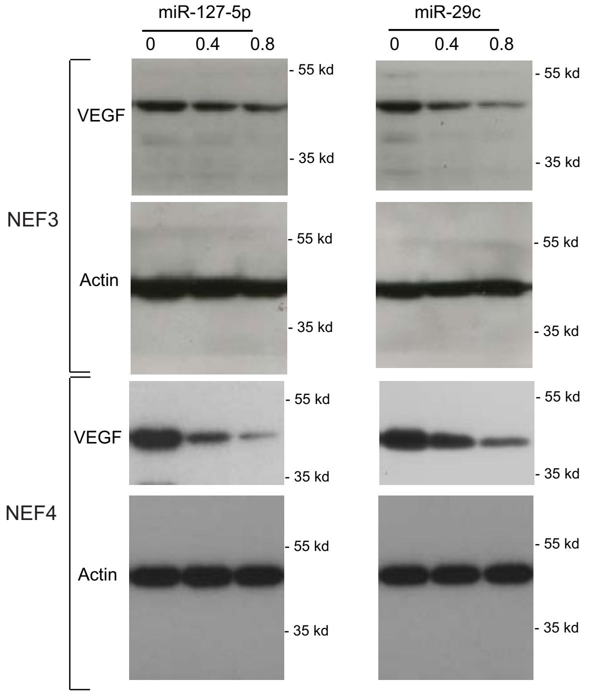

Supplementary Figure 9. Full blots of indicated figures.

Fig. 3d

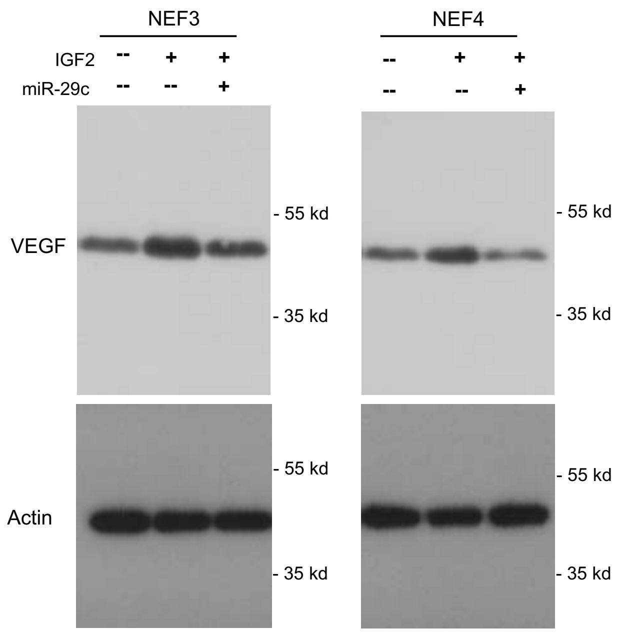

Fig. 3f

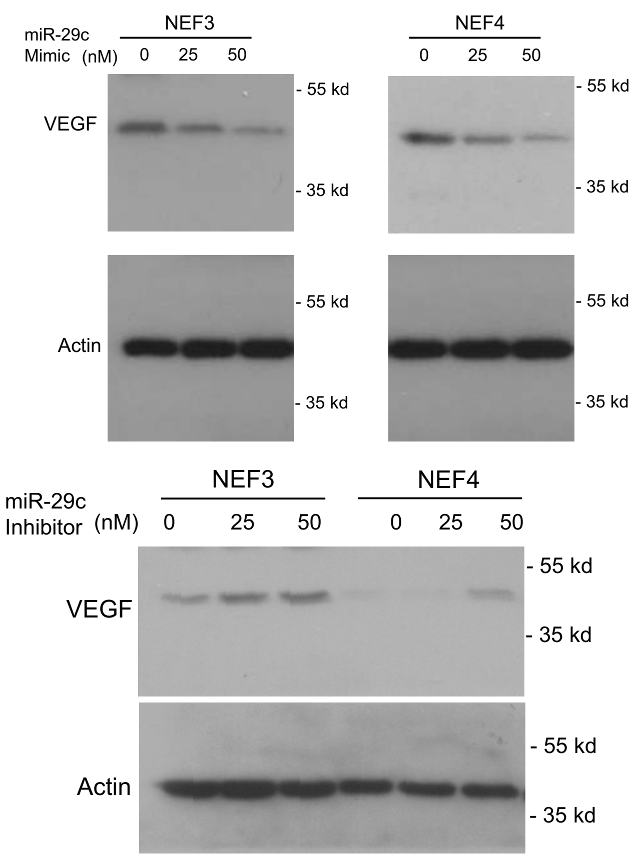

Fig. 4c

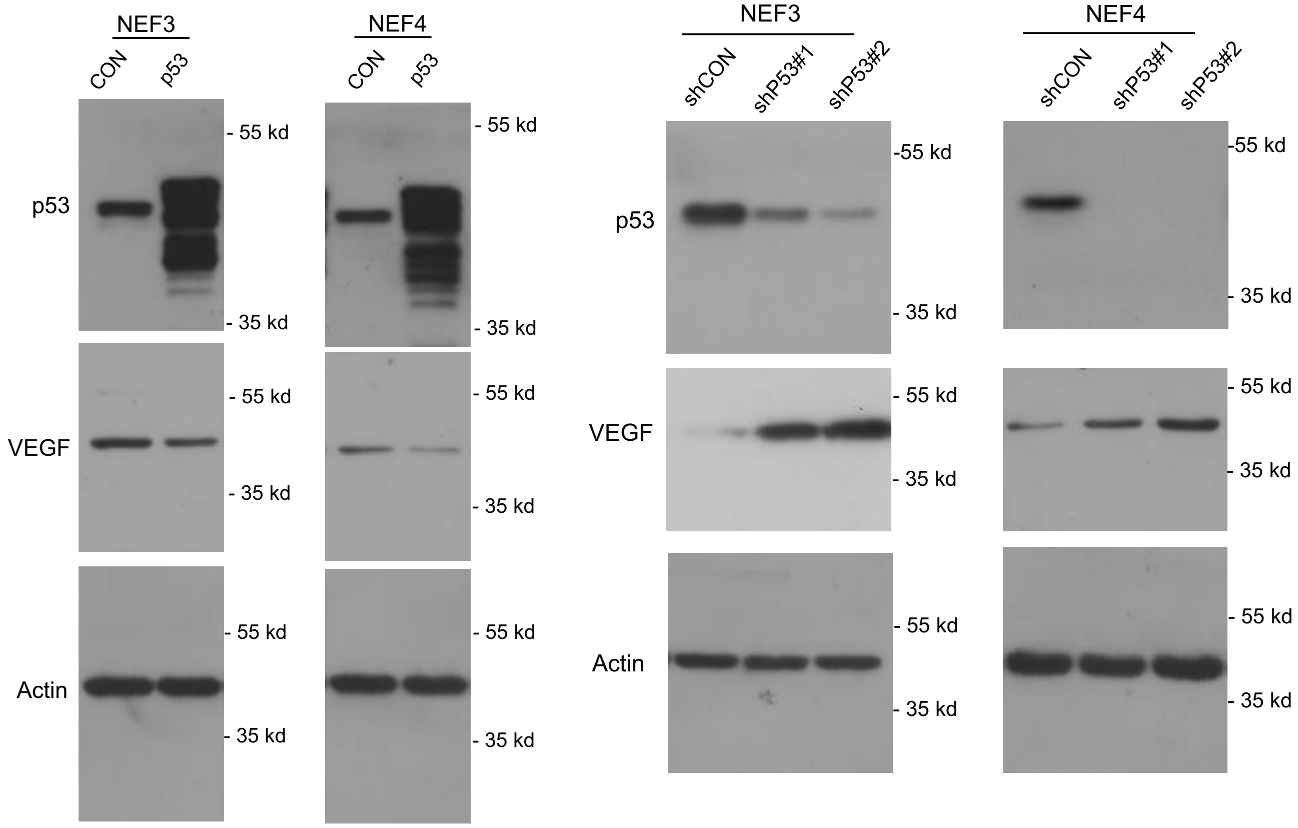

Supplementary Figure 10. Full blots of indicated figures.

Fig. 4f

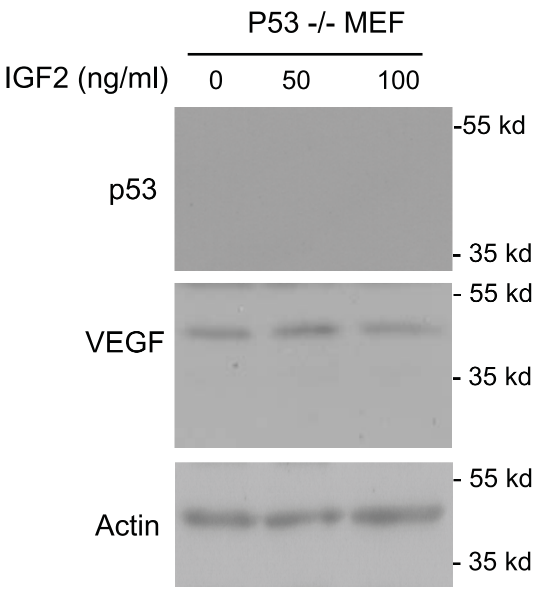

Fig. 4g

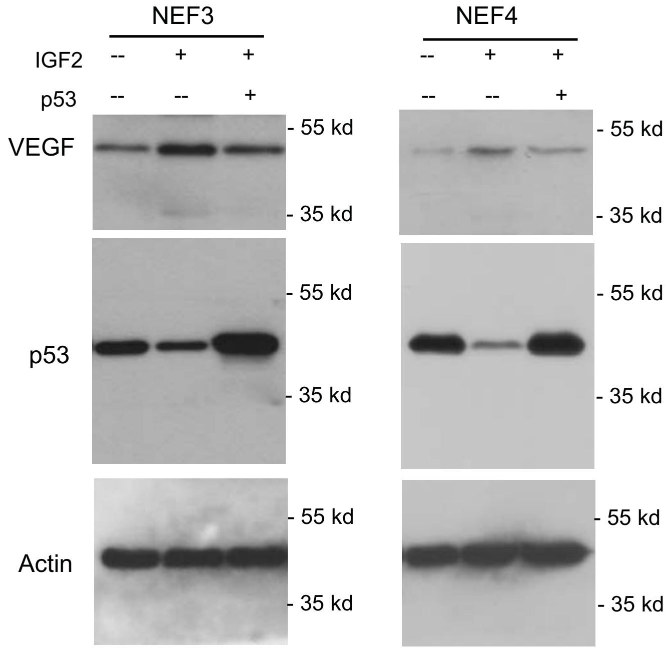

Supplementary Fig.1c

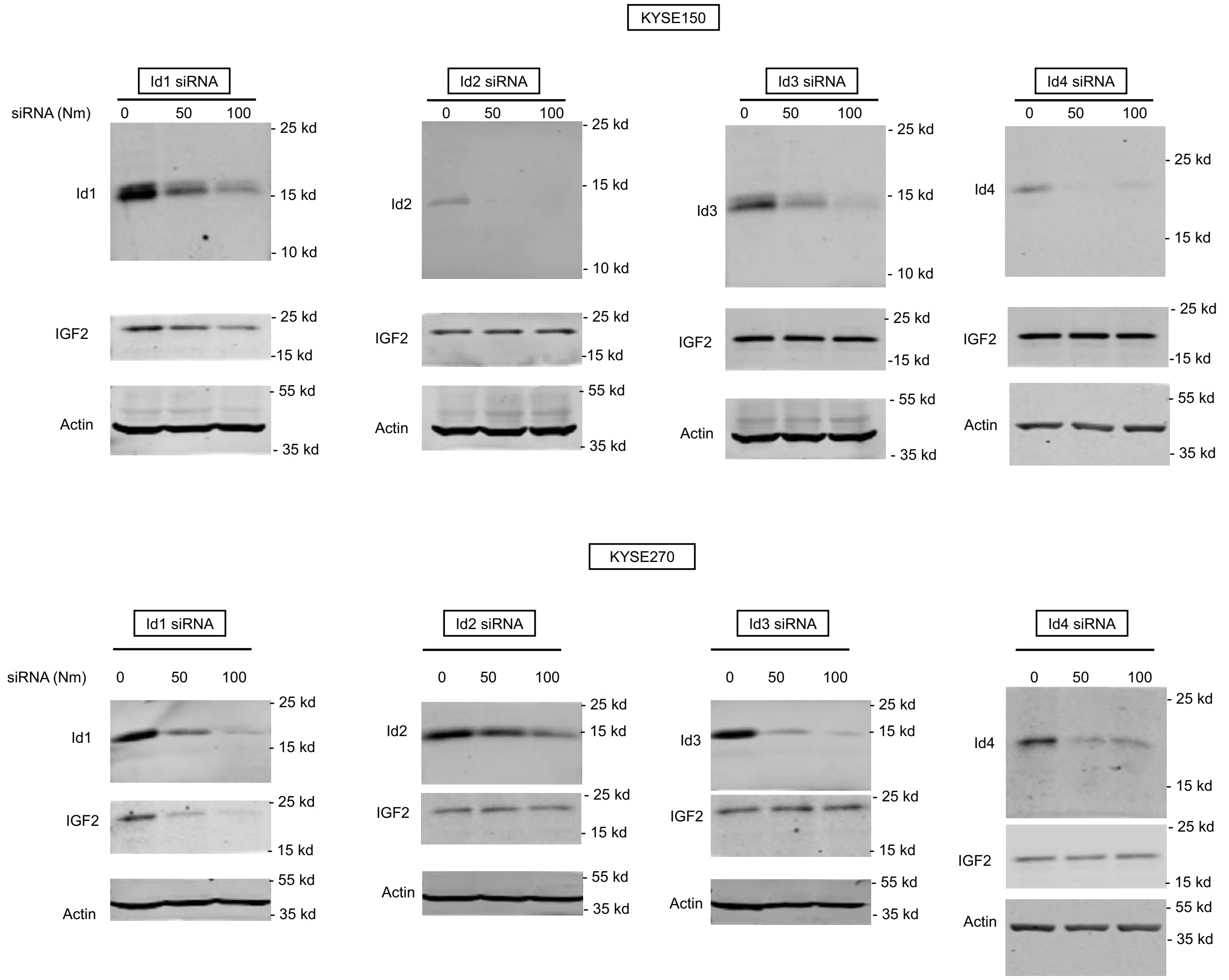

Supplementary Figure 11. Full blots of indicated figures.

Supplementary Fig. 1e

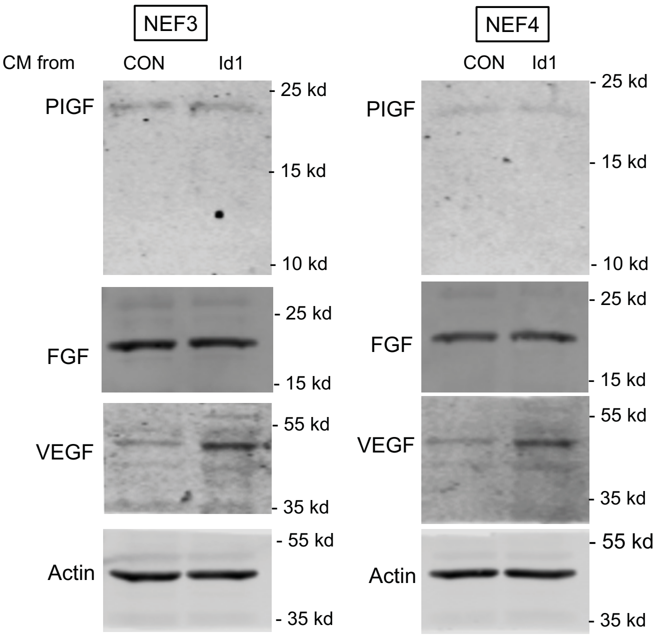

Supplementary Fig. 1h

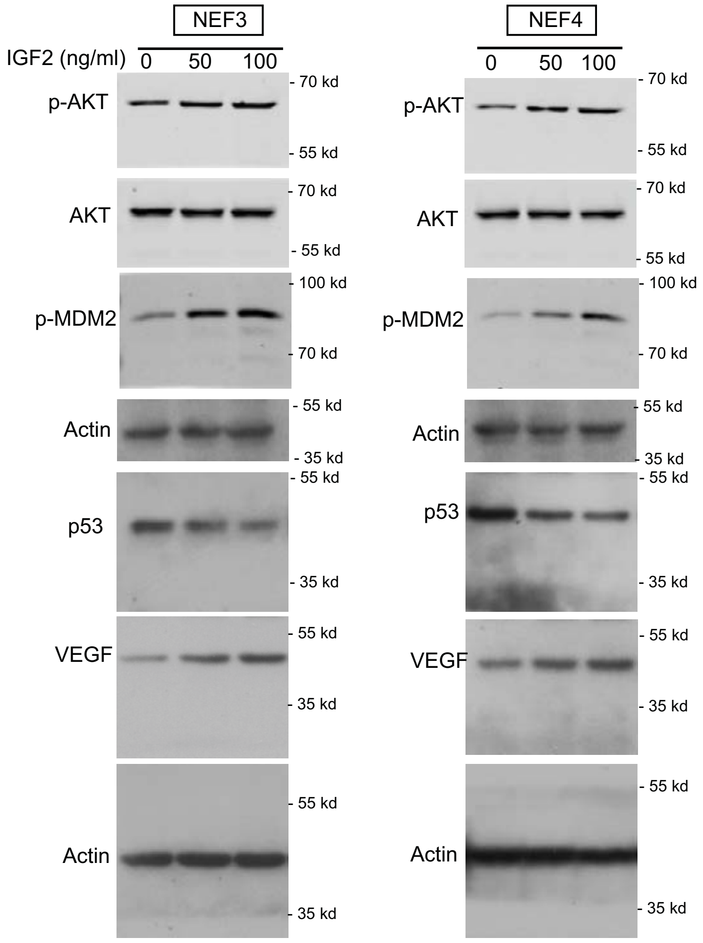

Supplementary Figure 12. Full blots of indicated figures.

Supplementary Table 1. Correlation between IGF2/VEGF expression levels and clinicopathological parameters in patients with ESCC.

Variable	n	IGF2 ^{Low}/VEGF ^{Low}	IGF2 ^{High}/VEGF ^{High}	*P* value [a]
**Age (years)**				
≤ 55	13	6	7	
≥ 55	51	26	25	1
**Gender**				
Female	16	8	8	
Male	48	24	24	1
**Histological grade**				
Poorly differentiated	14	8	6	
Moderately/well differentiated	48	23	25	0.762
**T-Stage**				
1/2	14	12	2	
3/4	50	20	30	**0.005**
**N-Stage**				
N0	32	18	14	
N1	32	14	18	0.454
**M-Stage**				
M0	58	32	26	
M1	6	0	6	**0.024**
**Pathologic stage**				
Stages I & II	26	18	8	
Stages III & IV	38	14	24	**0.021**

[a] Fisher's exact test. Statistical significance (*P* <0.05) is shown in bold.

Supplementary Table 2. Cox proportional hazard regression analyses for overall survival.

Clinical features	Univariate analysis		Multivariate analysis	
	HR (95 % CI)	*P* value	HR (95 % CI)	*P* value
Age	1.013 (0.545-1.880)	0.968	-	-
Gender	1.211(0.681-2.154)	0.515	-	-
Differentiation	1.453(0.811-2.602)	0.209	-	-
pT stage	2.342(1.203-4.562)	**0.012**	-	-
pN stage	1.748(1.044-2.926)	**0.034**	-	-
pM stage	3.455(1.414-8.439)	**0.007**	-	-
Pathological stage	2.476(1.438-4.263)	**0.001**	2.269(1.307-3.942)	**0.004**
IGF2/VEGF expression	1.459 (1.110-1.918)	**0.007**	1.372 (1.039-1.812)	**0.026**

HR Hazard ratio, CI Confidence interval

Statistical significance ($P < 0.05$) is shown in bold.

Supplementary Table 3. Primers used in RT-PCR for 40 putative miRNAs that bind with 3'UTR of VEGF.

miRNAs	Primer sequence
miR15a-5p	CTAGCAGCACATAATGGTTTGTGA
miR29c	GCTAGCACCATTTGAAATCGGTTA
miR103a	GCAGCATTGTACAGGGCTATGA
miR125a-3p	GTGAGGTTCTTGGGAGCCA
miR125a-5p	CCCTGAGACCCTTTAACCTGTGA
miR127-5p	CTGAAGCTCAGAGGGCTCTGAT
miR134-5p	GACTGGTTGACCAGAGGGGA
miR140-5p	GCCAGTGGTTTTACCCTATGGTAG
miR185-5p	GGAGAGAAAGGCAGTTCCTGAA
miR186-5p	CAAAGAATTCTCCTTTTGGGCT
miR199a-5p	AGTGTTCAGACTACCTGTTCA
miR200b	AATACTGCCTGGTAATGATGAA
miR205-5p	CTTCATTCCACCGGAGTCTGA
miR299	TATGTGGGATGGTAAACCGCTT
miR300	GCCTATACAAGGGCAGACTCTCTCT
miR329	GCAACACACCTGGTTAACCTCTTT
miR330-3p	GCACACGGCCTGCAGAGA
miR331-3p	CCCTGGGCCTATCCTAGAA
miR339-5p	GTCCTCCAGGAGCTCACG
miR361-5p	GCTTATCAGAATCTCCAGGGGTAC
miR374a	TTATAATACAACCTGATAAGTGAA
miR383-5p	GAGATCAGAAGGTGATTGTGGCT
miR410	GCGAATATAACACAGATGGCCTGTA
miR495	AACAAACATGGTGCACTTCTTA
miR503-5p	AGCGGGAACAGTTCTGCAG
miR516a-3p	TGCTTCCTTTCAGAGGGTAAA
miR593	GCTGTCTCTGCTGGGGTTTCT
miR874	TGGCCCGAGGGACCGA
miR939-5p	GCTGAGGCTCTGGGGGTG
miR3650	CGCAGGTGTGTCTGTAGAGTCC
miR3668	GCGCAATGTAGAGATTGATCAAAAT
miR3908	GCGAGCAATGTAGGTAGACTGTTT
miR4270	AGGGAGTCAGGGGAGGGC
miR4279	GCTCTCCTCCCGGCTTC
miR4455	CGCAGGGTGTGTGTGTTTTT
miR4481	CGGAGTGGGCTGGTGGTTA
miR4524a-5p	GATAGCAGCATGAACCTGTCTCA
miR4731-5p	GGGGGCCACATGAGTGTG
miR4742-3p	TCTGTATTCTCCTTTGCCTGCAG
miR4782-5p	GCCTTCTGGATATGAAGACAATCAA

Supplementary Table 4. Primer sequences used for testing p53 mutation.

Exon	Primer	Sequence (5′-3′)
exon1	Forward	CACAGCTCTGGCTTGCAGA
	Reverse	AGCGATTTTCCCGAGCTGA
exon2	Forward	AGCTGTCTCAGACACTGGCA
	Reverse	GAGCAGAAAGTCAGTCCCATG
exon3-4	Forward	AGACCTATGGAAACTGTGAGTGGA
	Reverse	GAAGCCTAAGGGTGAAGAGGA
exon5-6	Forward	CGCTAGTGGGTTGCAGGA
	Reverse	CACTGACAACCACCCTTAAC
exon7	Forward	CTGCTTGCCACAGGTCTC
	Reverse	TGGATGGGTAGTAGTATGGAAG
exon8-9	Forward	GTTGGGAGTAGATGGAGCCT
	Reverse	GGCATTTTGAGTGTTAGACTG
exon10	Forward	CTCAGGTACTGTGTATATACTTAC
	Reverse	ATACTACGTGGAGGCAAGAAT
exon11	Forward	TCCCGTTGTCCCAGCCTT
	Reverse	TAACCCTTAACTGCAAGAACAT

Supplementary Table 5. Primers used for generating pGL3-miR29c promoter mutations.

Primer	Sequence
BS1-F	5'-tcactcccttaccttccacataagaacagctcggacagacagat-3'
BS1-R	5'-atctgtctgtccgagctgttcttatgtggaaggtaagggagtga-3'
BS2-F	5'-gccctgtactaaatgttgcatcttgatttcttgagccatatgggct-3'
BS2-R	5'-agcccatatggctcaagaaatcaagatgcaacatttagtacagggc-3'
BS3-F	5'-tggccttgatggcactaaagaatgacccccagaaaggtcc-3'
BS3-R	5'-ggacctttctgggggtcattctttagtgccatcaaggcca-3'

Supplementary Table 6. Primer sequences used in ChIP-qPCR assay.

Primer	Sequence
ChIP-BS1-F	5'-tgtcttcctttcggcacttc -3'
ChIP-BS1-R	5'-tccaagttgtttgggtgtca -3'
ChIP-BS2-F	5'-gggctgccctgtactaaatg -3'
ChIP-BS2-R	5'-gtggtcagggtgaggaacat -3'
ChIP-BS3-F	5'-ggttgagcatgccaataaaga -3'
ChIP-BS3-R	5'-ggcaaatgggatttaagtaaccaga -3'
